# Local Orientation and the Evolution of Foraging: Changes in Decision Making Can Eliminate Evolutionary Trade-offs

**DOI:** 10.1371/journal.pcbi.1002186

**Published:** 2011-10-06

**Authors:** Daniel J. van der Post, Dirk Semmann

**Affiliations:** 1 Courant Research Center Evolution of Social Behaviour, Georg-August Universität Göttingen, Göttingen, Germany; 2 Institute of Artificial Intelligence, University of Groningen, The Netherlands; 3 Behavioural Ecology and Self-Organization, University of Groningen, The Netherlands; Medical College of Wisconsin, United States of America

## Abstract

Information processing is a major aspect of the evolution of animal behavior. In foraging, responsiveness to local feeding opportunities can generate patterns of behavior which reflect or “recognize patterns” in the environment beyond the perception of individuals. Theory on the evolution of behavior generally neglects such opportunity-based adaptation. Using a spatial individual-based model we study the role of opportunity-based adaptation in the evolution of foraging, and how it depends on local decision making. We compare two model variants which differ in the individual decision making that can evolve (restricted and extended model), and study the evolution of simple foraging behavior in environments where food is distributed either uniformly or in patches. We find that opportunity-based adaptation and the pattern recognition it generates, plays an important role in foraging success, particularly in patchy environments where one of the main challenges is “staying in patches”. In the restricted model this is achieved by genetic adaptation of move and search behavior, in light of a trade-off on within- and between-patch behavior. In the extended model this trade-off does not arise because decision making capabilities allow for differentiated behavioral patterns. As a consequence, it becomes possible for properties of movement to be specialized for detection of patches with more food, a larger scale information processing not present in the restricted model. Our results show that changes in decision making abilities can alter what kinds of pattern recognition are possible, eliminate an evolutionary trade-off and change the adaptive landscape.

## Author Summary

Animals differ in how they sense and process information obtained from the environment. An important part of this information processing is used to find food. In terms of foraging, local decision making determines how successful individuals are at finding food on longer timescales. Using an artificial-world model, we studied different kinds of decision making to understand how local information processing affects larger scale behavioral patterns and their evolution. We compared a restricted decision making (less memory) to extended decision making (more memory). We then compared the evolution of decision making and behavioral actions (moving and scanning for food) in patchy and uniform environments. Our results show that with restricted decision making individuals face a trade-off in the patchy environment: they try to stay in patches by not moving forward too far, but to do so they sacrifice how fast they travel between patches. With extended decision making this trade-off completely disappears because decision making allows moving forward to be avoided in patches. Instead moving forward can be used exclusively for faster traveling between patches and for selecting bigger patches. Our results show how changes in local decision making can significantly alter what evolutionary forces are faced and can eliminate evolutionary trade-offs.

## Introduction

The evolution of behavior is to a large extent the evolution of information processing [1]–[4]. On short timescales individuals respond to local information in the environment. For instance in foraging, a basic local information processing is that animals detect food, turn and move to food, and eat. On the long term this generates behavioral patterns. The latter shapes how individual behavior relates to patterns in the environment (e.g. resource distributions) and affects aspects of Darwinian fitness (e.g. foraging success). At present it is poorly known how local information processing mechanisms (e.g. cognition) determine larger scale pattern detection and evolve [3], [5]–[8]. Here we study the evolution of local information processing and orientation to the environment, and its relation to environmental pattern detection.

In evolutionary theory on foraging, the focus is often on how well individuals match (fitness relevant) patterns in the environment. In optimal search theory (OST) the main focus has been on what kinds of random turning strategies optimize search [9]–[11]. A second focus has been on the value of alternating between intensive searching, once a food patch is found, to extensive search when food has not been found for a while, using combinations of correlated random walks differing in turning rates [12]. Simulations show that such switching between search strategies can enhance foraging efficiency because it concentrates search effort in the right places (i.e. it allows patches to be “detected”), so called area-concentrated search. This is true for models in which “continuous” patchy environments are assumed [12], [13], where resource items are only locally detectable, but aggregated on a scale that is beyond the perception of individuals, as apposed to models in which discrete and fully detectable patches are assumed (e.g. the marginal value theorem [14]).

Random-walk models have been used to statistically characterize animal movement trajectories, including bi-modal search patterns similar to area-concentrated search [15], [16]. However, such model fitting does not necessarily reveal underlying movement mechanisms [6], [17]. Interaction with, and orientation to, the external environment can generate similar movement patterns as those generated by internal turning strategies [6], [17], [18]. Moreover, Benhamou showed that local orientation via memory of where an individual last found a food item, can further improve foraging efficiency relative to “random” area-restricted search without such memory [19], indicating the adaptive value of reacting to external cues. However, like the random-walk search models, an important assumption is that food is detected and consumed on the same range. Instead, if food can be detected beyond the range at which it can be eaten (as is often the case), an animal will be able to approach foraging opportunities from some distance via direct visual cues. This is probably one of the most simple ways through which animals can orientate themselves relative to food. Important is that such opportunity-based adaptation (or responsiveness) stands in direct relation to feeding opportunities in the environment. Therefore, on longer timescales, behavioral patterns emerge that are “a reflection of complexity in the environment” [20].

**Figure 1:**
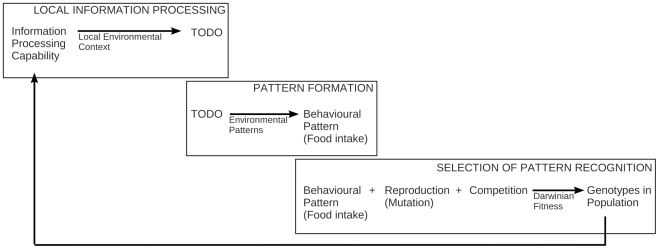
Illustration of multi-level information processing. *Local information processing* depends on an individual’s genotype (information processing capabilities) and local environment context, generating TODO (behavioral adaptation to local opportunities). *Pattern formation* then arises through TODO on larger spatial and temporal scales beyond the perception of individuals in relation to patterns in the environment. *Selection of pattern recognizing* genotypes arises through differences in reproductive rates (Darwinian fitness) of individuals that vary in their pattern formation and compete over food. Thus short arrows indicate information processing and the information being processed (arrow label). The long arrow indicates how genotypes selection feeds back on information processing capabilities present in the population.

To conceptualize how interaction of individuals with the environment can structure behavior, Hogeweg and Hesper [21] coined the TODO principle. This envisages behavior as multi-scale information processing [22], [23] (see Figure 1): (i) TODO: individuals behaviorally adapt to local opportunities by “doing what there is to do”, and (ii) Pattern formation and detection: behavioral patterns self-organize on larger spatio-temporal scales through the continual feedback between behavior and local environmental contexts (This use of the term “information processing” differs from that in behavioral ecology where it generally refers only to individual-level behavioral flexibility, often specifically in relation to energy-dependent behavioral choices). A simplistic example of TODO is that as food density declines individuals end up moving more and eating less, because there is no opportunity to eat. As such, the environment is like a “behavioral template” to which individuals can respond, allowing individuals to effectively “detect” patterns of opportunities in the environment beyond their own perception.

In order to fit models to movement data and elucidate underlying mechanisms, requires a thorough understanding of how both internal and external structuring of behavior can generate foraging patterns. This can be done using pattern oriented modeling [24] and other multi-level modeling approaches [25], where model fits are evaluated based on patterns on multiple levels: small scale movement decisions, mesoscale patterns such as trajectories and space use and more global patterns such as population distributions. The requirement of fitting models to multiple levels places the focus on the mechanisms that generate the inter-relation between small-scale processes and patterns on larger scales. A thorough understanding of how small scale behavior interactions generate behavioral patterns through TODO could be an important contribution to such modeling approaches.

Essentially, TODO and the longer term behavioral patterns it generates, come to expression (in models) when individuals interact with the environment and need to make behavioral decisions based on local information. In this light, Hogeweg [26] showed that foragers with simple TODO rules could forage much more efficiently than those with much more complicated rules. This was because foragers with simple rules could react to local opportunities and therefore automatically adapt to larger-scale patterns in the environment (i.e. generalize their behavior). More counter-intuitive and complex behavioral patterns emerge in models with more detailed environmental structure and multiple types of behavior. Examples include “self-structuring” explanations for social dynamics in bumblebee colonies [27], grouping patterns in chimpanzees [28], diet learning and cultural inheritance in group foragers [29], [30].

At present, the role of pattern recognition through TODO is most likely underestimated in most approaches to the evolution of foraging behavior. For instance in OST the simple orientation mechanism of turning and moving to food is generally not included. Moreover, behavior is usually assumed to be continuous in that movement, search and food consumption occur in parallel (although a trade-off between movement speed and search accuracy is often assumed [12]). Decision-making is therefore restricted to changes in direction. However, if movement, scanning for food and eating are at least partially mutually exclusive, then individuals must decide about what to do next (e.g. search again at a certain location, or move on). Such foraging behavior can be referred to as pause-travel [31], or intermittent search [7]. Here we focus on local orientation towards food in such a setting where individuals must make decisions, and study the role of TODO in the evolution of simple foraging behavior. We ask: how does local information processing evolve in order to determine how individuals “do what there is to do”? More specifically, how does the responsiveness and orientation of individuals to feeding opportunities in the environment evolve in light of the larger spatio-temporal pattern recognition that this generates?

To address this question, we study the evolution of foraging behavior in a model with individuals that have to choose amongst alternative behavioral actions according to information they obtain through searching. This happens in a spatial environment with patchy and uniform patterns of feeding opportunities. To address how local information processing (sensing and decision making) affects information processing on larger spatio-temporal scales (pattern recognition and genetic adaptation, see Figure 1), we compare the evolution of decision making and properties of behavioral actions in two model variants. In a “restricted” model we limit information individuals can remember and use relative to an “extended” model. The comparison across environments is used to understand evolutionary adaptation to prevailing ecological conditions (patchy or uniform). The comparison across models (restricted versus extended) is used to understand how differences in the evolutionary freedom (or constraints) for evolving decision making affect evolution. This has similarities to artificial neural network approaches to the evolution of behavior, where behavior is not predefined, but emerges from neural architecture and learning processes [32]–[35]. Such models have been used to show, for instance, that risk-averse foraging can emerge as a side-effect of an evolved reinforcement learning process [33]. In our case there is no learning, but the “architecture” of decision making can evolve such that non-predefined behavior can evolve. Therefore we do not prespecify a selection function, but only define that inter-birth intervals decrease with increased food intake, and allow natural selection to arise from competition in a world with finite resources. We then study how Darwinian fitness arises as an emergent property of how micro-scale interactions generate longer-term behavioral patterns. Thus, we study evolution as the interplay of information processing on multiple timescales (Figure 1), based on bioinformatic (processes) theory [22], [23], [36]–[38].

Using this approach, we show that local information processing and opportunity-based adaptation can play a significant role in detecting patterns of resources in the environment, and the evolution of foraging. In particular, we find that the differences in decision making capabilities affect how individuals interact with the environment (TODO), and this can alleviate evolutionary trade-offs and allows for novel pattern recognition specializations.

## Materials and Methods

### Model

Our model incorporates (i) individual foragers and (ii) a 2-dimensional environment with resource items in either a patchy or uniform distribution, adapted from van der Post and Hogeweg [29]. Individuals have a decision making algorithm which determines the sequence and context dependency of the following behavioral actions: MOVE, FOODSCAN, MOVETOFOOD and EAT. Each of these behavioral actions has specific properties (such as distances, angles etc). Our model is event-based, which means that actions take time. When individuals complete an action they choose a new one. The individual with the shortest time to complete its action is next to choose a new action.

We study two model variants (“restricted” and “extended”) which differ in the type of decision making algorithm that can evolve. Both the parameters of the decision making algorithm and the details of behavior are “genes” which change through mutation. This generates genetic variation, which may result in differences in foraging efficiency and rates of reproduction. Natural selection then arises from resource competition. For a full list of model parameters please see Table 1 and 2. Next we discuss the model in more detail.

**Table 1:** Non-evolvable parameters (the boundary conditions for evolution).

Category	Parameter / description	Value	Units
Timescale	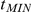 (minimal duration)	10	sec
	day	720	min
	year	365	days
Environment	grid unit	1	m
	field size	5.66 x 5.66	km
Resources	renewal interval	1	year
	density	0.535	items per 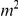
	detection distance	2	m
	detection probability	1	per sec per 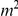
	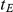 (handling time)	10	sec
	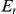 (energy)	2	units
Patches	number	8000	patches
	patch radius	20	m
	resources per patch	2500	items
Individuals	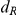 (individual reach)	0.9	m
	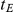	10	sec
	maximum speed	0.1	m/sec
	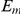 (metabolism)	1	units/min
	minimal energy	0	units
	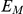 (maximum energy)	100000	units
	birth requirement	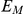	units
	birth energy costs	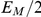	units
	offspring energy	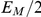	units
	death rate	0.1	per year
	maximum age	10	years
	mutation rate	0.05	

**Table 2:** Evolvable parameters of individuals.

Category	Parameter / description	St. dev.	Min	Max	Units
Durations	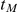 (move duration)	0.2(2)	0.167	1.99	min
	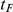 (food scan duration)	0.2(2)	0.167	1.99	min
Distances	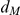 (move distance)	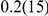	0.0	-	m
	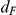 (food scan distance)	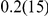	0.0	-	m
Angles	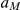 (turning angle)	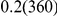	0.0	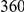	degrees
	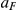 (food scan angle)	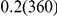	0.0	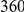	degrees
Probabilities	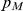 (repeat move)	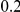	0.0	-	
(restricted model)	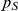 (repeat food scan)	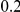	0.0	-	
(extended model)	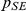 (repeat food scan after eat)	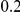	0.0	-	
(extended model)	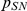 (repeat food scan after nofood)	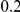	0.0	-	
(extended model)	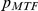 (move to food)	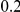	0.0	-	

The standard deviation of mutation is scaled (0.2(x)) relative to what was considered a reasonable range for the parameter. The maximum of durations was imposed due to how the model was programmed, but was high enough not to affect the results.

### Environment

Our environment is 5660 by 5660 lattice, where grid points are scaled to be 1 meter apart, giving 32,035,600 grid points (32.035 km squared). This size was chosen to support a population size (about 100–150 individuals). This was the minimal population where: (i) parameters evolved, (ii) the population is self-sustaining, and (iii) simulations are completed in a reasonable time span. It also ensures that individuals need to move through space to find food, survive and reproduce. Resource items were placed on grid points. Resource items appeared at fixed, but randomly assigned time points within a year, and remained there until eaten. If eaten the resource item was depleted, and appeared again at its fixed time point in the year. Days are 720 minutes (12 hours of “daylight”) and years are 365 days (262800 minutes).

We implement a patchy and a uniform environment, where we keep the total number of food items constant and only vary the resource distribution. In the patchy environment we placed 8000 patches, each with about 2500 items depending on overlap of randomly positioned patches. Each patch is a circle with a radius of 20 meters. Within this circle, 2 resource items are placed at each grid point. All resource items in a patch appear at the same time point, and different patches appear at random fixed times in the year. In the uniform environment resources are placed with probability 0.535 per grid location to match the total number of resources placed in the patchy environment (17150000 items). In the uniform environment, resource items appear at randomly assigned fixed times throughout the year.

### Decision making

The restricted and extended model differ in the decision making that can evolve. Figures 1a and b show the basic decision making algorithms: the behavioral actions that are possible (ovals) and in the case of FOODSCAN, the information this provides (rectangles). Arrows indicate what can be done next, or what information is obtained (after FOODSCAN), and an individuals last action (+ information obtained) represents its “state” (or memory). EAT and MOVETOFOOD can only occur after food is detected. EAT occurs when food is detected in range, otherwise individuals first MOVETOFOOD (MTF) and then EAT. Without any information about food, individuals can either MOVE or do FOODSCAN. As a starting condition, we set these to alternate so that individuals always do FOODSCAN after MOVE and vice versa.

**Figure 2:**
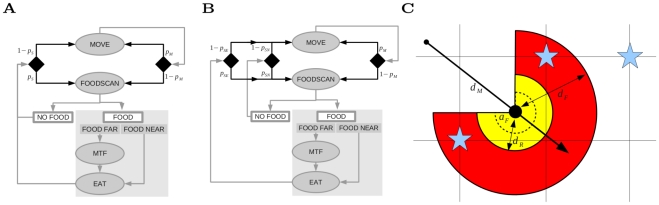
Illustration of decision making and behavioral actions. Decision making algorithms of (a) restricted and (b) extended models. Ovals: behavior actions (MTF  =  move to food), Squares: information acquired from the environment, Diamonds: decision points. Arrows indicate the sequence of actions, decision points, and information. Gray arrows: fixed, Black arrow: evolvable (in the restricted model this depends on 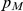 and 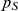. In the extended model 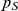 is split into two: 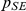 and 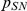 creating an extra decision point). The shaded gray square indicates fixed behavior that occurs in the “FOOD” context. (c) Visual representation of foraging: 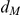 is the distance covered with MOVE (solid line), followed by a FOODSCAN (red) of angle 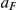 about forward direction (thickest arrow) over distance 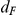. This can detect food (blue stars) placed on a grid. If food is beyond reach 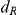 then the individual will MOVETOFOOD (to the closest star detected) before EAT.

To allow decision making to evolve we define parameters which determine the probability of moving again after MOVE (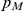) and scanning again after FOODSCAN (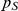) (Figure 2a), searching again after EAT (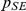), or searching again after NO FOOD (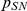) (Figure 2b, see also Table 2). This is indicated by decision points (black diamonds) after MOVE, NO FOOD and EAT, where arrows split. For each of these probabilities, the alternative decision has a probability of 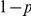. For the restricted model we only allow 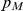 and 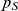 to evolve, where 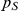 is a general probability to do FOODSCAN again, irrespective of whether individuals have eaten or did not find food (Figure 2a). Thus in the restricted model, the probability to do FOODSCAN again after EAT or after NO FOOD, is determined by the same parameter (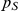). For the extended model we allow 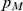, 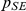, and 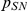 to evolve (Figure 2b), where 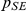, and 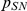 can be seen as context dependent forms of 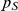. In the extended model, the probability to do FOODSCAN again after EAT or after NO FOOD, can therefore evolve independently. Thus, in the restricted model individuals cannot remember and make use of the additional information “just ate” or “didn’t find food” to determine the probability to do FOODSCAN again, while in the extended model they can. Moreover, in the restricted model, we assumed individuals always MOVETOFOOD when food is out of reach. In the extended model we allowed this probability (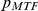) to evolve, and it always evolved to 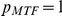 (see section 2 in Text S1 and Figure S1).

### Behavioral actions

The parameters of specific behavioral actions determine how individuals move and sense their environment (see Figure 2c). Unless stated otherwise, we allow all these parameters to evolve:

- **MOVE.** Individuals step with distance 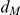, duration 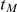 and turn with angle 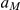 about their current direction. An individual’s speed is 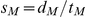, where maximum speed is limited to 360 meters per hour, in order to scale all behaviors to the same minimal time step (10 seconds). Individuals move in a continuous space and can move in any direction. In principle individuals can occupy the same exact point, and therefore can be in the same grid cell in the environment. When individuals reach the edge of the environment, they choose a random direction. Note that the turning angle can also evolve and occurs during every MOVE event, but randomly to the left or right. This is a simplified version of turning angles studied elsewhere [7].
- **FOODSCAN.** Resource items are searched for 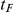 seconds within an area defined by radius 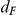 and angle 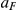 about an individual’s forward heading (Figure 2c, red area). Resources are only detectable if within 2 meters (the environmental constraints on detection). Moreover, the probability to detect a given resource item 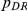 depends on how long individuals spend scanning per meter squared:
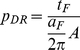
(1)

where 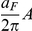 is the area scanned (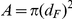), and where 1 second of scanning for 1 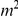 gives 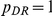. The closest detected item is chosen for consumption. If there are multiple items equally close, a random closest item is chosen. This scanning algorithm therefore represents the case where individuals eat the first item they find. Note also that we assume that MOVE and FOODSCAN cannot occur at the same time, and thus we focus pause-travel foraging [31] or “intermittent search” behavior [7].

- **MOVETOFOOD.** Individuals move to within half of their reach (0.45 meters) from a chosen food item, taking 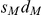 seconds. Individuals may attain a new heading when turning to move towards food. We chose an individual’s reach (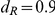, nonevolvable) to be less than its maximal range for detecting resources ( = 2 meters) because this appears reasonable for many animals.
- **EAT.** Individuals spend 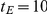 seconds eating a resource item (nonevolvable).

### Energy, survival and reproduction

Individuals gain energy through food (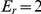 energy units per item) which is added to their energy store 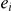 (with a maximum: 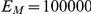). To survive, individuals must have energy (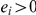), which means energy intake must compensate basal metabolism (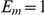, which is subtracted from 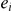 every minute). Because resources become locally depleted individuals must move to eat. We do not add explicit movement costs, but time spent moving cannot be spent eating. Individuals reproduce when 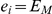. Energy is then halved and the other half goes to a single offspring. The time taken to get back to 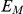 defines a birth interval. Individuals with shorter birth intervals achieve greater lifetime reproductive success. Individuals can die with a probability of 0.1 per year, and can reach a maximum age of 10 years. This adds some stochasticity in survival and limits lifespans to 10 years. Since resources are limited in the environment, the population grows until the reproduction is at replacement rate (carrying capacity).

Our model requires that the population is viable in relation to resource availability, thus energy and life-history parameters are chosen such that at low population sizes individuals can definitely gain sufficient energy to reproduce. Moreover, to focus on movement and foraging in differently patterned environments, we set the energy required to give birth in relation to energy per food time, and the density of food items in space, such that individuals have to move to and forage from many food patches and experience the full scale of environmental patterns during a reproductive cycle (i.e. they cannot complete reproductive cycles within a single patch). Lifespan is set to allow multiple reproductive events per individual. We expect most parameter combinations that satisfy these qualitative relationships (see section 1 in Text S1 for more detail), to give similar results.

### Mutation

When individuals reproduce, the parameters of decision making and behavioral actions are inherited by offspring, with a probability of mutation of 0.05 per gene (this rate of mutation was chosen after observing that natural selection lead to consistent evolutionary change with increases in foraging efficiency). We allow all action durations, distances and angles to evolve except 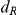 and 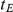. The mutation “step” is defined by drawing the parameter value from a normal distribution with the mother’s parameter value as mean and standard deviation scaled to about 20% of the range of values that is relevant for that parameter (see Table 2). Moreover, in order to keep simulations running fast enough, we limited the minimal action duration to 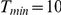 seconds. Most mutations are close the mother’s parameter value, but larger jumps are possible. This was chosen to make evolution of parameters possible without predefining their ranges.

### Initial conditions

We cannot predict what parameter settings are viable and take a “zero” state (all parameters zero) as initial condition. To make sure the population does not die out initially, we use a birth algorithm in which the non-viable population is maintained at a minimum of 10 individuals, and let it evolve to a viable state. During this time, if the population drops below this minimum then an individual is chosen to reproduce according to a probability (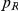) relative to its energy (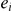):
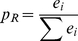
(2)


Energy costs of reproduction and energy of offspring as the same as before. Once the population grows above 10 individuals and becomes viable, this algorithm is not used anymore. At this point the population grows to carrying capacity and becomes stable.

### Simulations and analysis

For our study we used the following types of simulations:

1. **Evolutionary simulations.** We ran 10 large-scale evolutionary simulations for 1000 years, starting from the “zero” initial conditions and with mutation on genes. We do this for both the restricted and extended model in both patchy and uniform environments. We analyze evolutionary simulations by conducting ancestor traces, backtracing lineages from the final population to the beginning of the evolutionary simulations. Through this method we reveal lineages that survive to the end of the simulation (see section 2 in Text S1 and Figure S1). Thus we obtain lineages representing the evolution of parameters in our model. We take parameters of ancestors between year 800 and 900 to represent “evolved genotypes” (those at year 1000 include recent mutants, which possibly have not been under selection for long enough). These simulations provided our core results, which were used as inputs into the two types of simulations described below.
2. **Ecological simulations.** To study evolved genotypes, we compare them in detail in shorter non-evolutionary simulations (no reproduction, death or mutation). Because competition arises in our model through resource depletion, we compare different evolved genotypes *together* in the same simulations to determine how they forage *relative to each other*. For speed reasons we use a smaller field (4000 by 4000 m) with a fixed population of 65 individuals and study foraging behavior. We run the simulation until we have 100 samples of a year of foraging for each evolved genotype. For this size field, 65 individuals is the carrying capacity scaled relative to the full field (125 individuals) and we therefore study behavior at the same resource density as in the evolutionary simulations (roughly 0.01–0.05 items per 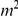 due to depletion).
3. **Characterizing the adaptive landscape.** Evolutionary pressures in our multi-dimensional evolutionary space (8 parameters) could be quite complex, depending on how the different parameters inter-relate. Moreover these inter-relationships could change with the change in decision making and environment. To study this we conducted simulations where we varied 2 given parameters across individuals, while keeping other parameters on evolved values (no reproduction, death or mutation), for each evolved genotype in its respective environment. We could not run separate simulations for each parameter combination because in our case fitness differences between individuals only come to expression through resource depletion. Moreover, when conducting this analysis we introduce many individuals that forage poorly, thus affecting foraging competition and reducing resource depletion. We therefore raise the population until resource depletion approaches that normal for evolving populations (as stated above). Thus we obtain a local characterization of the adaptive landscape about the evolved genotypes allowing inter-relations between parameters to be revealed.

## Results

### What evolves?

**Figure 3:**
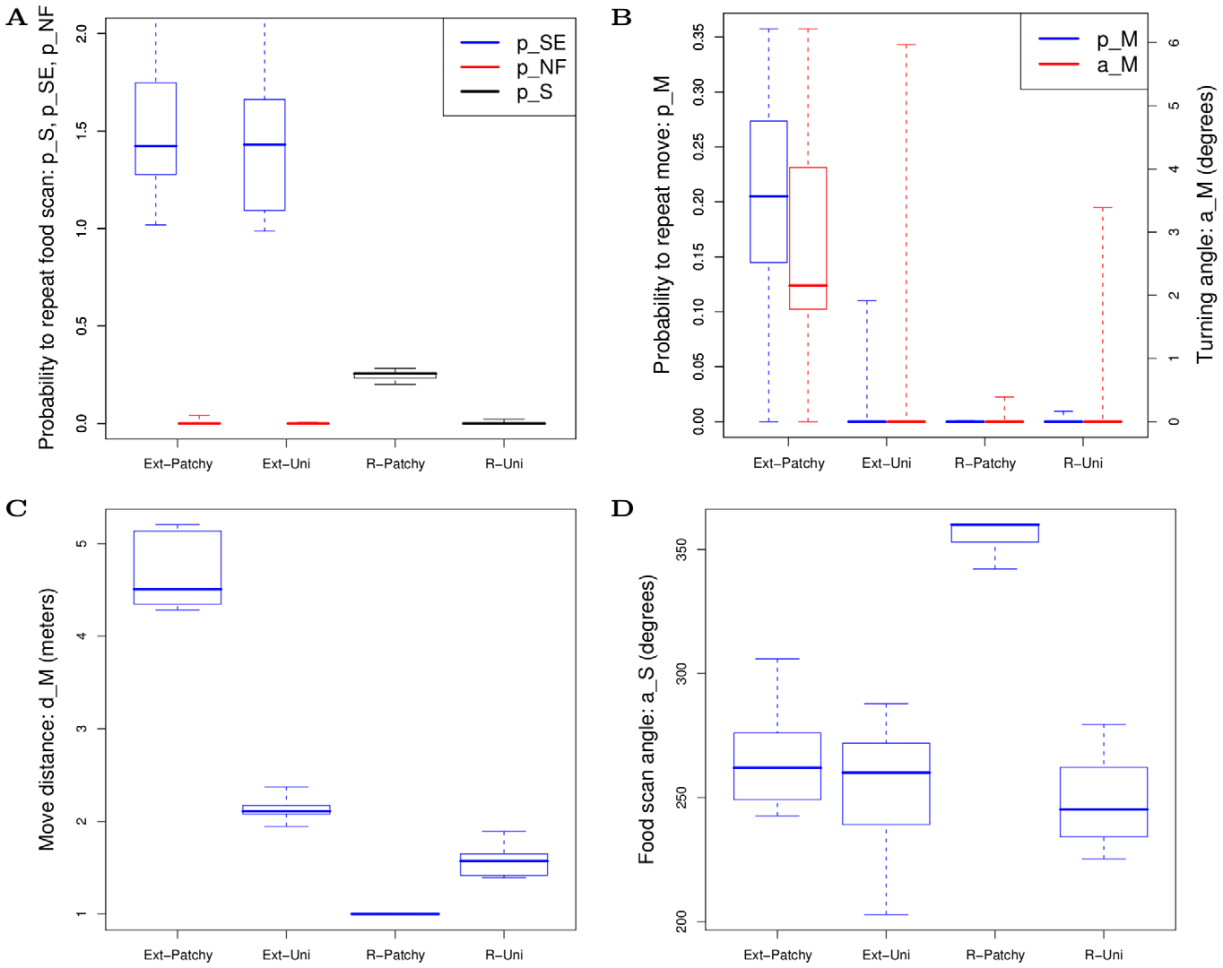
Evolved foraging parameters in restricted and extended model in patchy and uniform environments. (a) probability to scan for food again: 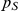 (restricted model), 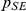 and 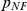 (extended model, left and right respectively), (b) probability to move again (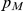) and turning angle (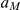) (left and right respectively), (c) move distance (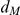), (d) food scan angle (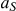). Box plots show data from year 800 to 900 from 10 ancestor traces in each case. Box plots show, median, upper and lower quartile, and whiskers show max and minimum values.

**Figure 4:**
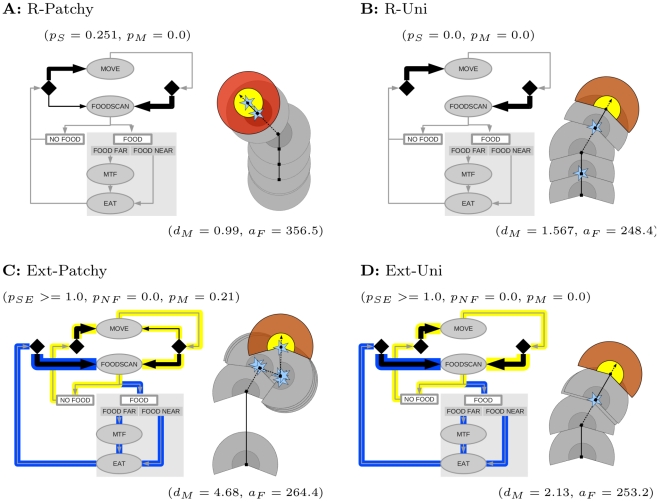
Evolved decision making and behavioral actions. For each specialist we show the decision making algorithm (left) and an illustration of foraging behavior (right). **Decision making:** in the extended model (c and d) a decision making evolves which clearly differentiates behavior in FOOD and NON-FOOD contexts. This is illustrated with the yellow loop (“always MOVE after NO FOOD”) and the blue loop (“always SEARCH after EAT (FOOD)”). Switching between loops occurs when food is detected or not. In contrast in the restricted model (a and b), some repeated SEARCH only evolves in the patchy environment (a: arrow from EAT and NO FOOD to SEARCH), but organizing behavior into separate loops is not possible. Repeated search does not evolve in the uniform environment (b). Shapes and arrows as in Figure 2. **Behavioral actions:** most striking is the full circular search (shaded areas), short MOVE distance (solid lines) and large overlap of search areas of R-Patchy (a), in contrast to the long MOVE distance, smaller search angle and smaller overlap in search in Ext-Patchy (c). More subtle is the shorter move distance and larger overlap in search areas of R-Uni (b) compared to Ext-Uni (d). Other details: gray and red  =  previous and latest SEARCH, dashed lines  =  MOVETOFOOD, blue stars  =  EAT, dashed lines with arrow  =  heading, inner circles/pie sections  =  REACH (gray is previous, yellow is present).

We find that in both models the population evolves to environment specific attractors. We refer to these evolved states as “specialists”: uniform specialists in the uniform environment, and patch specialists in the patchy environment. These four specialists differ from each other and these differences depend on the following parameters: (i) probabilities to SEARCH again (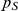, 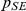, 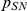), (ii) probability to MOVE again (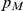), (iii) MOVE distance (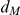), (iv) turning angle (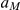), and (v) FOODSCAN angle (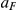) (see Figure 3). For ease of reference we name the specialists and summarize their distinguishing features as follows (illustrated in Figure 4). Parameter values shown are means of ancestor traces between year 800 and 900 (see also Table S1):

- **R-Patchy** (restricted model patchy): has some repeated food scanning (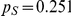, Figure 3a), the shortest move distance (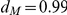, Figure 3c), and the largest food scan angle (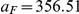 degrees, Figure 3d).
- **R-Uni** (restricted model uniform): has no repeated search (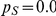, Figure 3a) and has the second shortest move distance (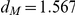, Figure 3c).
- **Ext-Patchy** (extended model patch): always repeats food scan after finding food (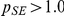), and never repeats food scan after not finding food (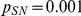, Figure 3a, blue and orange respectively), is the only specialist to repeat MOVE (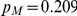, Figure 3b, blue) and turn while moving (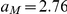 degree, Figure 3b, orange), and has the longest move distance (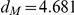, Figure 3c).
- **Ext-Uni** (extended model uniform): has the same food search probabilities as Ext-Patchy (Figure 3a), but does not evolve repeated MOVE or turning (Figure 3b, blue and orange respectively). It moves 1.35 times further than R-Uni (Figure 3c; this difference is significant: Wilcoxon rank sum test, 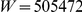, 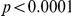. For Ext-Uni: 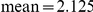; 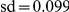; 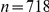. For R-Uni: 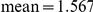; 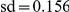; 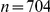).

Further analysis revealed that variation of both probability to repeat move (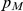) and turning angles (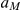) did not impact food intake significantly. For both parameters we found that evolved values result from evolutionary drift because of a very flat adaptive landscape (for more detail see Text S1 section 2 and Figure S1 and Text S1 section 4 and Figure S3 and S4). Moreover, other parameters did not differ between specialists: durations evolved to minimal values (see section 2 in Text S1 and Figure S1) and food scan range (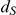) converged to between 2–2.5 meters (see sections 2 and 3 in Text S1 and Figure S2). From here on we focus on those parameters that generated differences in foraging efficiency between the specialists, namely: 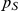, 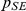, 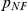, 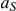 and 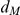. We use the means of evolved parameter values to characterize each specialist (see Table S1 for a complete list of average evolved parameter values).

### Behavioural implications: decision making and actions

The values of the evolved decision making parameters mean that in the extended model decision making evolves to: **always do FOODSCAN after EAT, always MOVE after NO FOOD** (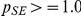 and 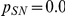, Figure 4c and d). This generates a clear differentiation of behavior in food and non-food contexts (Figure 4c and d, blue and yellow loops respectively). Thus in a food context individuals continue to do FOODSCAN until they no longer find food (blue loop). This generates efficient FOODSCAN - EAT - FOODSCAN - EAT sequences and allows systematic depletion of resources at a given location. During this time any movement is via MOVETOFOOD when food is out of range, always towards food. Only when no more food is found do individuals MOVE. Thus in a “no food” context, individuals switch behavior and no longer repeat FOODSCAN (yellow loop).

In the restricted model only the patch specialist (R-Patchy) has a certain degree of repeated scanning for food (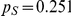, Figure 4a). However this happens equally after EAT and NO FOOD, because differentiating behavior relative to FOOD and NOFOOD is not possible. This specialist therefore can only to a certain extent avoid MOVE in the presence of food, and is more limited in generating time efficient FOODSCAN-EAT sequences and to only MOVETOFOOD when food is beyond REACH. In contrast the uniform specialist (R-Uni) of the restricted model **never repeats FOODSCAN** (Figure 4b). It only searches once per location and generates MOVE - FOODSCAN - EAT or MOVE - FOODSCAN - MOVETOFOOD - EAT sequences.

For behavioral actions the most obvious difference between the specialists is that between the patch specialists of the different models (illustrated in Figure 4a and c). R-Patchy’s maximum FOODSCAN angle in combination with its short move distance leads to a behavioral pattern with a large overlap in areas searched after each MOVE. In contrast, Ext-Patchy’s smaller FOODSCAN angle with long move distance generates a pattern with long distances in which it does not scan, followed by food directed movement when food is detected. The difference between the uniform specialists is more subtle (Figure 4b and d). The shorter MOVE of R-Uni leads to considerable overlap in areas scanned after each MOVE. Ext-Uni’s longer MOVE leads to hardly any overlap in areas scanned after each MOVE.

### Ecological implications: behavioral patterns and foraging efficiency

**Figure 5:**
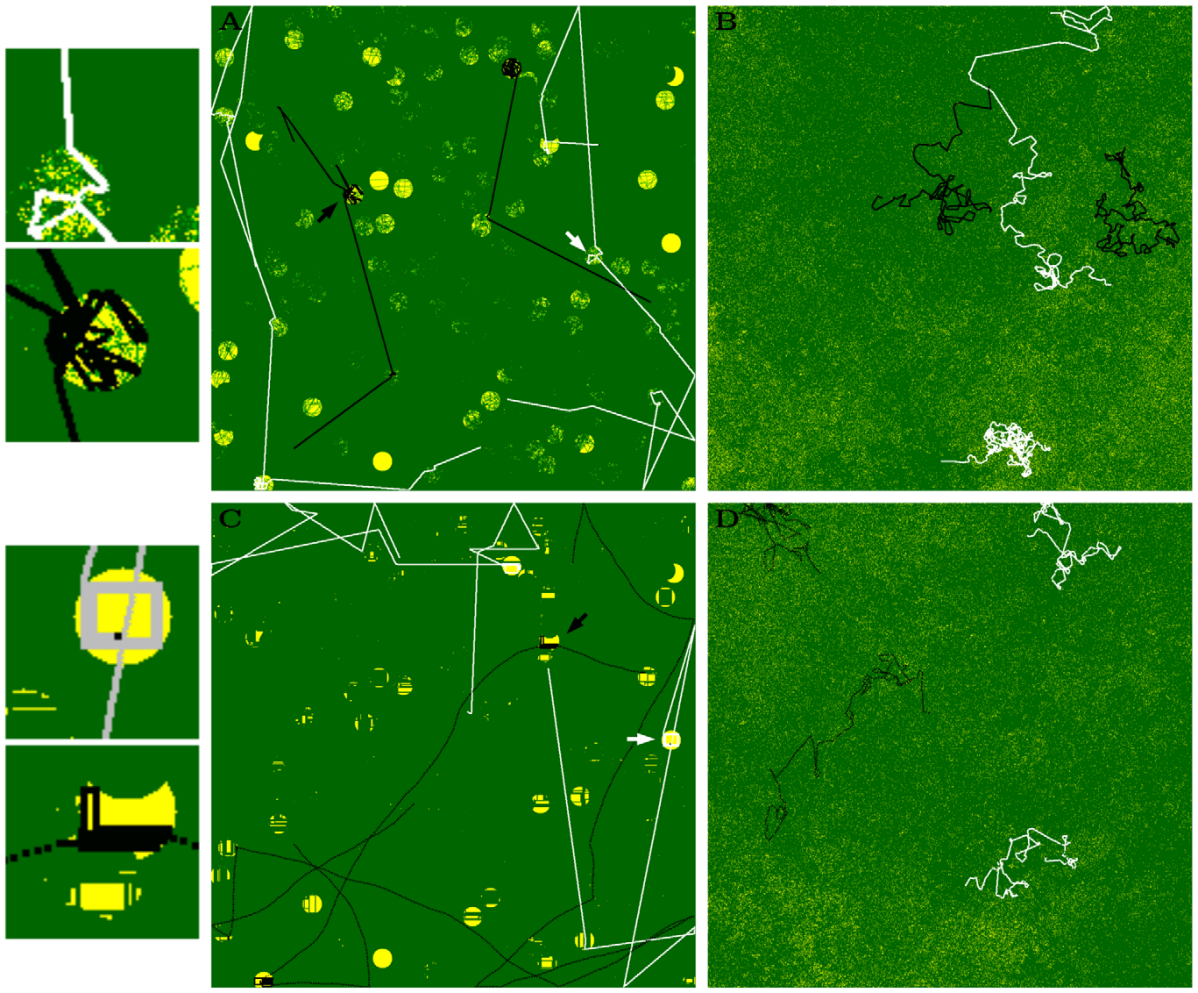
Movement trajectories generated by TODO. Both uniform (white) and patch (black) specialists adapt flexibly to both environments (left and right). This is true for the restricted (top) and extended (bottom) model. The basic TODO is MOVE when there is no food, turning to MOVETOFOOD and stopping to EAT. This generates “random walks” in the uniform environment and bi-modal between- and within-patch movement in the patchy environment. These movement patterns reflect opportunities for feeding in the environment. Within-patch behavior (indicated by arrows) is shown for both restricted (top) and extended (bottom) models in more detail in the smaller figures on the left. Dark green: background. Yellow: resources. Field size is 1 by 1 km.

To qualitatively reveal larger-scale behavioral patterns, we visualize the movement trajectories of all evolved specialists in both environments using ecological simulations (Figure 5) . Most striking is that it is difficult to distinguish between the specialists in the same environment, because they all adapt flexibly to both environments, whether they evolved there or not. This is because all specialists are responsive to opportunities in the environment, and have the same basic TODO (“do what there is to do”): move when there is no food, turn and move to food when out or reach, and stop to eat. In the uniform environment this generates random-walk-like patterns reflecting the random encounters with food. In the patchy environment TODO generates a bi-modal pattern of straight movements between patches and frequent turning and remaining localized for some time within patches. Thus irrespective of genetic adaptations, through (automatic) opportunity-based adaptation all specialists are able to generalize their behavior to an environment in which they did not evolve.

**Figure 6:**
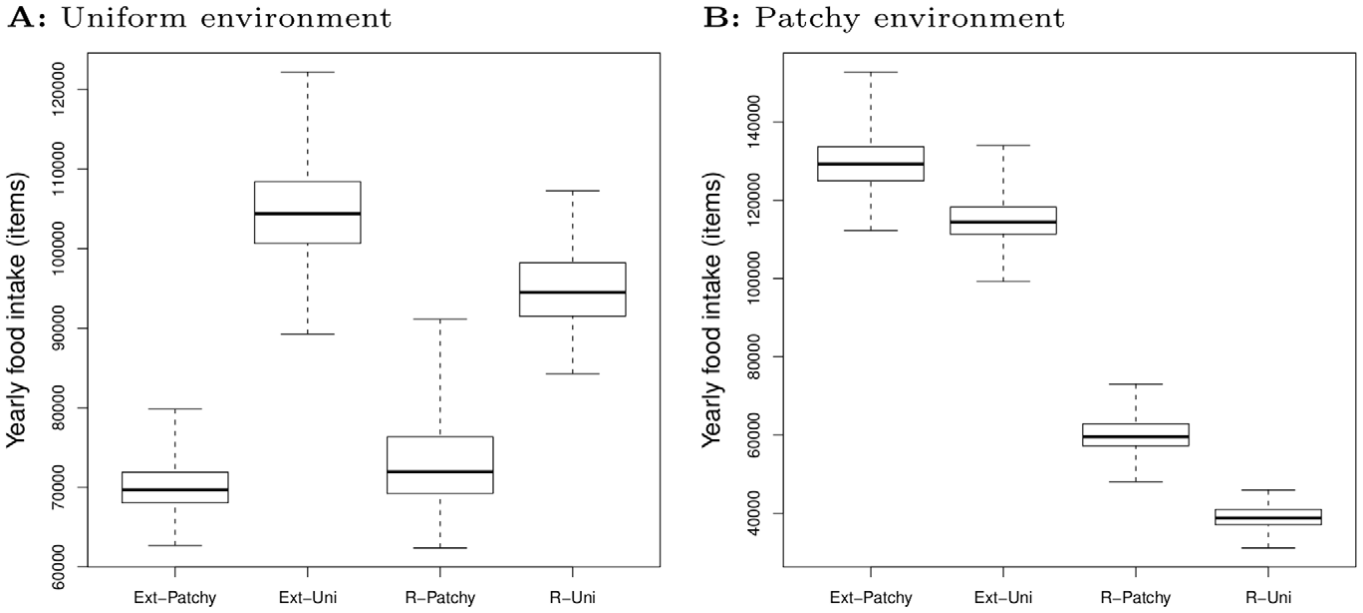
Comparison of foraging efficiency of evolved specialists. Yearly food intake in (a) uniform and (b) patchy environments. Box plots represent medians, upper and lower quartile and max and minimum (n = 100).

The large-scale behavioral patterns of individuals reflect patterns of feeding opportunities in the environment (patchy or uniform). The more accurate this reflection, the better individuals “detect” resource patterns, and this affects their foraging success. An individual’s genotype determines how it responds to opportunities in the environment, and we find that the genetic adaptations of specialists increase their foraging success relative to the environment they evolved in (Figure 6). Overall, differences in food intake rates of evolved specialists, as measured in ecological simulations, are as follows:

- Uniform environment:

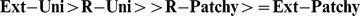
 (Figure 6a).

- Patchy environment:

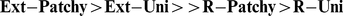
 (Figure 6b).

where 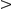 represents a minor difference, and 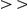 a large difference.

In both environments, specialists from the extended model are the most successful foragers. Interestingly, Ext-Uni is not only the best forager in the uniform environment, but the second best in the patchy environment. In the uniform environment, Ext-Uni has about 9% greater food intake than R-Uni (this difference is significant: Wilcoxon rank sum test, 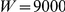, 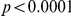. For Ext-Uni: 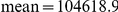; 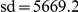; 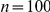. For R-Uni: 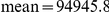; 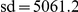; 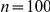). In the patchy environment, Ext-Uni has on average about 11% lower food intake than Ext-Patchy (this difference is significant: Wilcoxon rank sum test, 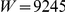, 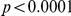. For Ext-Uni: 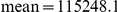; 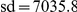; 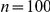. For R-Patchy: 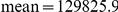; 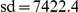; 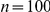). However, Ext-Uni has nearly 2 times greater food intake than R-Patchy, even though it did not evolve in the patchy environment (unlike R-Patchy). In contrast, Ext-Patchy is the least successful forager in the uniform environment, although average food intake is only about 3% lower than R-Patchy (but this difference is significant: Wilcoxon rank sum test, 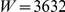, 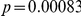. For Ext-Patchy: 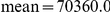; 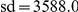; 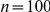. For R-Patchy: 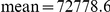; 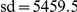; 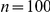). Overall, differences in the patchy environment are greater (2 fold versus a 1.5 fold maximum difference in the uniform environment), indicating more room for specialization. To understand these results we look in detail at how changes in decision making and behavioral actions affect food intake.

### Adaptive landscapes and evolutionary attractors

The difference in decision making capabilities of the two models has a profound effect on the evolutionary landscape. This is most clear in the patchy environment, where the enhanced information use in the extended model allows a trade-off on within- and between-patch behavior to be eliminated. Therefore, while we find that evolved parameters in both patch specialists reflect a tendency to maximize food intake by (i) trying to stay in patches, and (ii) minimizing inter-patch travel, how this is achieved depends on how the underlying decision making capabilities shape the evolutionary landscape.

**Figure 7:**
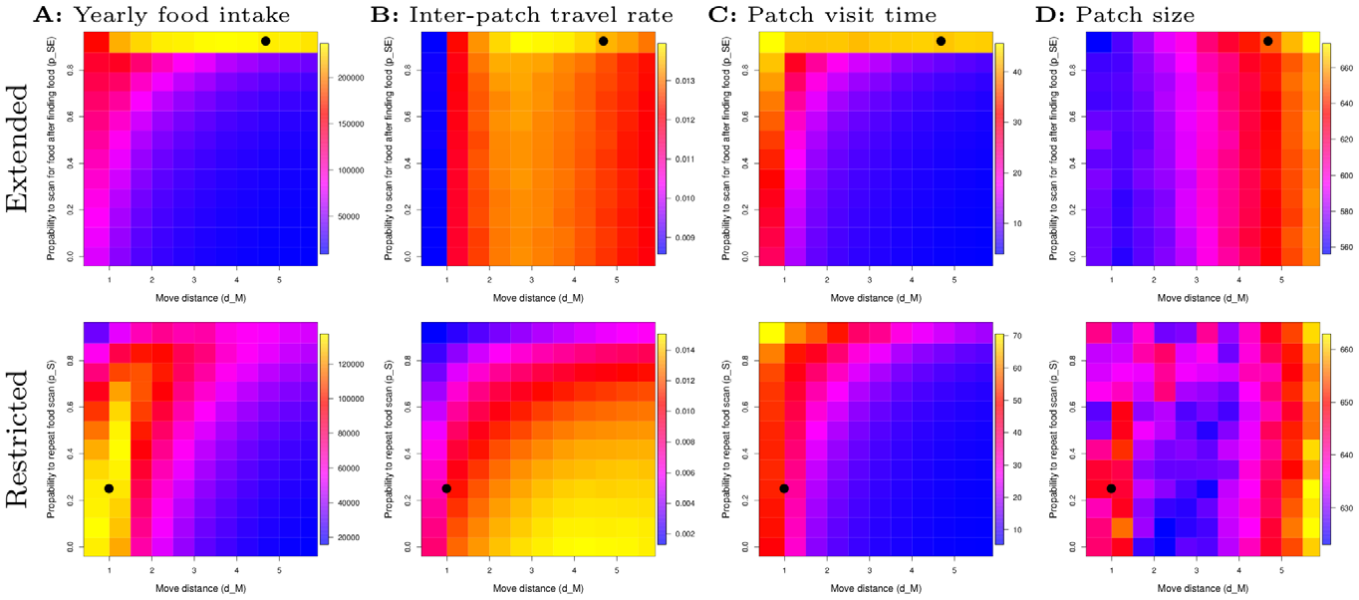
Local adaptive landscape in patchy environment of (i) probability to scan for food again (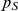 and 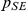) and (ii) move distance (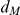). Top: extended model. Bottom: restricted model. From left to right: yearly food intake (fitness), inter-patch travel rate (inverse inter-patch travel time), patch visit time, patch size. Values are normalized within one figure, and a gradient from dark blue to yellow, via green and red, indicates increasing values. Each grid point is the average of 100 samples of a year of foraging. Black circles indicate average evolved parameter values.

This is most clearly illustrated with a local adaptive landscape characterization around the evolutionary attractors relative to the probability to search again (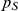 and 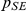) and move distance (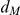). We consider how parameters affect yearly food intake (“fitness”), and how this depends on inter-patch travel, patch visits time (i.e. how much they manage to eat in a patch) and size of patches visited (Figure 7).

The comparison between the extended model (top) and the restricted model (bottom) reveals a significant shift in the location of the adaptive peak (Figure 7a top and bottom, yellow zone), which coincides with evolved parameter values (indicated by black circles). In the restricted model we can understand the location of the adaptive peak (and evolved parameters) in terms of a trade-off between inter-patch travel rate, and patch visit times. As one increases, the other declines (compare Figure 7b and c bottom row). This is because in order to stay in patches (and find food), individuals need short move distances and repeated food scans, otherwise they prematurely leave the patch. However, this slows down inter-patch travel with redundant search. The evolutionary attractor is therefore located where interpatch-travel time and intrapatch-travel time are such that food intake is maximized (Figure 7a, bottom). As a result R-Patchy has the slowest inter-patch travel of all specialists (see section 5 in Text S1 and Figure S5). Moreover, this is also why R-Patchy has such a large food scan angle, because this allows it to “turn back” when it inadvertently leaves a patch (see section 3 in Text S1 and Figure S2), and why it does not evolve repeated moving (see section 4 in Text S1 and Figure S3).

In the extended model this trade-off does not arise. Here decision making allows differentiation of behavior: food scanning is only repeated after eating and does not occur during inter-patch travel (no food encountered). Repeated food scanning can therefore evolve to maximal values, which allows individuals to move systematically from one food item to the next within patches via MOVETOFOOD. This leads to longer patch visit times (Figure 7c top) and enhanced patch depletion. Unlike in the restricted model, MOVE is now used purely for inter-patch travel. Move distance (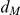) is then freed from the trade-off between inter- and intra-patch travel because it no longer affects patch visit times. The enhanced decision making in the extended model therefore eliminates the trade-off, allowing both extended model specialists to be more efficient than R-patchy.

As a consequence of the trade-off disappearing, move distance evolves to much longer distances (Figure 3c) because this allows individuals to bias foraging to larger patches (Figure 7d top). (Note that while we implement patches of a fixed size, partial depletion of patches generates smaller patches.) In fact there are two feedbacks which affect that individuals bias their patch visiting to larger patches: (i) by extending patch visiting times, an individual visits on average larger patches longer, and (ii) by reduced scanning for food while moving during inter-patch travel (i.e. due longer move distances) individuals are less sensitive to each food item on their way. Thus they are more likely to find food and stop moving when local resource densities are higher. Effectively this allows individuals to “select” larger patches. Therefore, for the same time spent traveling, Ext-Patchy manages to find on average larger patches and eat more than Ext-Uni (see section 5 in Text S1 and Figure S5 for more detail). Long move distances also generate more neutrality for repeated move and turning angles, allowing them to evolve (see section 4 in Text S1 and Figure S3 and S4).

**Figure 8:**
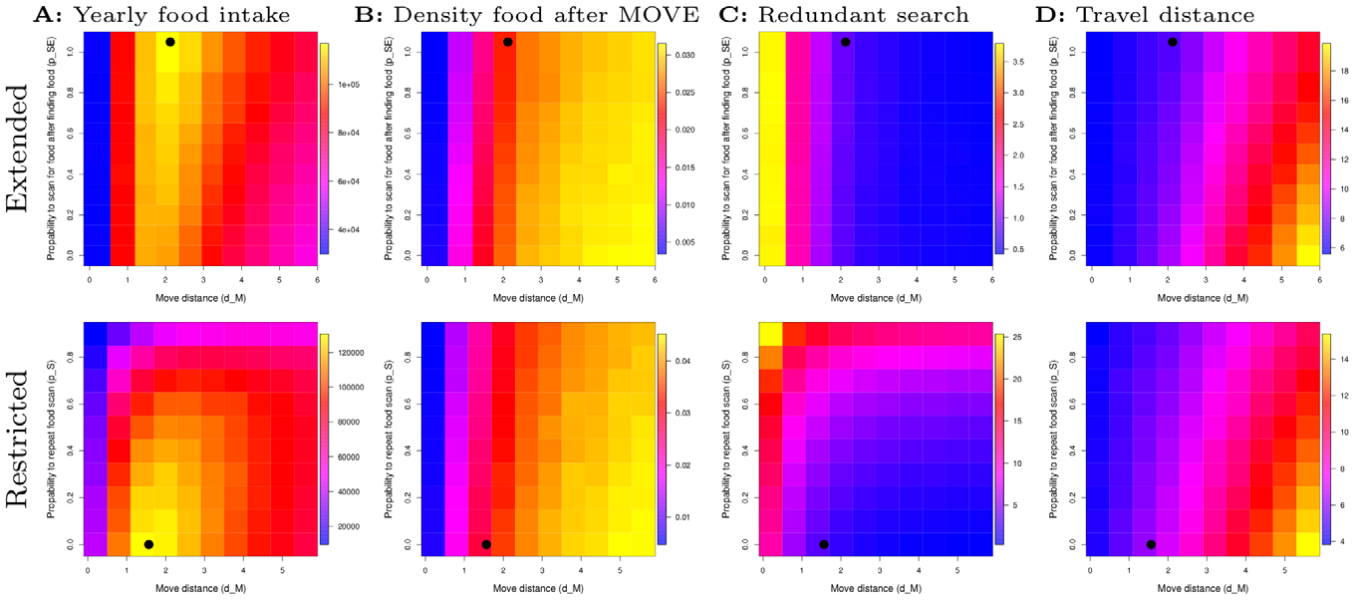
Local adaptive landscape in uniform environment of (i) probability to scan for food again (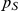 and 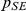) and (ii) move distance (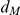). Top: extended model. Bottom: restricted model. From left to right: yearly food intake (fitness), inter-patch travel rate (inverse inter-patch travel time), patch visit time, patch size. Values are normalized within one figure, and a gradient from dark blue to yellow, via green and red, indicates increasing values. Each grid point is the average of 100 samples of a year of foraging. Black circles indicate average evolved parameter values.

For the uniform specialists we also find a difference between the extended and restricted model. Both specialists tend to maximize food intake by (i) not wasting time searching depleted areas, and (ii) not moving too far and skipping food items on the way. However, in the extended model food intake peaks at maximal repeated search after finding food, while in the restricted model food intake peaks at minimal repeated search and slightly shorter move distances (Figure 8a, top and bottom respectively).

In both cases, local depletion of food causes that individuals who move further during MOVE, find a greater average density of food during their next food scan (Figure 8b). However, the further individuals move the longer they travel between food items (Figure 8d). By repeating food scans, travel between food items can be reduced because several food items can be eaten at a given location (Figure 8d, see interaction between 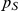 and 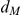). However, for the restricted model, redundant food scanning (when no food is found) rises quickly with repeated food scanning (Figure 8c, bottom), because FOODSCAN also happens after not finding food. The best option is therefore not to repeat food scanning (and therefore not systematically deplete a given location), but not move too far, as to not miss undepleted food items on the way. In the extended model, repeated food scanning only occurs after eating, and redundant food scanning is avoided, unless individuals do not move far enough (Figure 8c, top). Here the best option is therefore to always repeat food scans, systematically deplete a given location and move somewhat further than in the restricted model, to avoid a larger depleted area.

Overall Ex-Uni is more efficient than R-Uni (Figure 6a). Both are more efficient than patch specialists in the uniform environment (Figure 6a), because these either have too much redundant overlap in search (R-Patchy, due to repeated search) or skip too many resources on the way (Ext-Patchy, due to long MOVE distance) (see section 5 in Text S1 and Figure S6 for more detail).

### Other environments

To further evaluate our results we studied evolution in an intermediate patchy environment (twice as many patches, but half the density of resources) and a mixed environment (half resources uniform half patchy, only with extended model). In the intermediate patchy environment we find that foraging parameters evolve to be qualitatively the same as our main patchy environment both in the extended (parameter averages are: 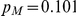, 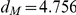, 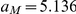, 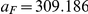) and restricted model (parameter averages are: 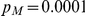, 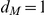, 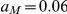, 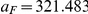). This indicates that the behavioral adaptations in the patchy environment are relatively robust to this change in patchiness although the difference in search angles is less pronounced. It is however likely that much smaller patches would select for smaller move distances, because in the mixed environment we find that the extended model evolves to be most similar to Ext-Uni (parameter averages are: 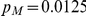, 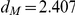, 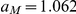, 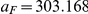). This makes sense given that Ext-Patchy does much worse than Ext-Uni in the uniform environment compared to the performance of Ext-Uni relative to Ext-Patchy in the patchy environment (see Figure 6). Selection for generalizability in more heterogeneous environments will therefore probably lead to Ext-Uni type genotypes.

## Discussion

Our results show how responsiveness to opportunities in the environment, and the behavioral pattern formation this generates on longer timescales, can play a significant role in the evolution of foraging behavior. This is because the behavioral pattern formation is also a type of pattern “recognition”, i.e. a larger-scale information processing. To illustrate this more explicitly we consider foraging in the patchy environment. When an individual hits a patch, it only detects a single food item. From that position it can find a neighboring food item and move towards it. Through this feedback between sensing and responding to the environment, the individual effectively uses the spatial auto-correlation of positions of food items as a template to move through the patch (see Figure 5). Effectively, “by doing what there is to do” on a very local scale, the individual generates a behavioral pattern that reflects the position of the patch. Because the behavioral pattern determines food intake, it has value in terms of rates of reproduction. Through natural selection, information about rates of reproduction is processed, effectively selecting behavioral patterns that better match, or “recognize” patterns of feeding opportunities in the environment. This drives changes in the population frequencies of genotypes which define how individuals “do what there is to do”. As such, the evolution of local information processing occurs through information processing on multiple timescales: (i) responses to local opportunities, (ii) formation of behavioral patterns and (iii) natural selection based on behavioral pattern formation (as illustrated in Figure 1).

Our comparison of extended and restricted decision making reveals that decision making capabilities determine the specificity with which individuals can respond to opportunities in the environment and the types and accuracy of pattern recognition. Specificity is greater in the extended model, where individuals could remember and use the information “found food here last scan” or “did not find food last scan”. This allows the context-dependent responses “always scan for food after eat” and “always move after no food found” to evolve, and behavioral differentiation between food and non-food contexts (Figure 4). In the restricted model this was not possible and individuals were less able to characterize local contexts when deciding to scan again: they only had the information that they had scanned, but not what the outcome was. The behavioral differentiation between food and non-food in the extended model allows systematic depletion of resources at a given location, and a more accurate recognition of patterns in the environment (e.g. patches), which is why the extended use of information evolves. Moreover, because larger-scale environmental patterns are spatial arrangements of local opportunities, greater specificity relative to local opportunities via TODO leads to greater behavioral generalization across environments. As a consequence, Ext-Uni performs better than R-Patchy in the patchy environment, even though only the latter evolved there. This reveals that generalization capacity, which leads to behavioral flexibility on the large-scale, can evolve in individuals via “hard wired” TODO tuned to local variation in foraging opportunities.

The differentiation of behavior in food and non-food contexts in the extended model significantly changes the adaptive landscape (selection pressures) and possibilities for larger scale pattern recognition (Figure 7). In the restricted model, in order to repeat FOODSCAN in patches, individuals also had to repeat scanning for food when moving between patches. Moreover, MOVE could not be avoided in patches. This lead to a trade-off on within- and between-patch behavior (Figure 7b and c, bottom). In the extended model, due to behavioral differentiation, MOVE is only used in non-food contexts, and repeated scanning only occurs in a food context. As a consequence there is no trade-off (Figure 7b and c, top), and MOVE is dissociated from selection pressures in the food context. Instead MOVE can become specialized for inter-patch travel, generating a refinement in larger-scale pattern recognition in order to detect a sub-pattern: patches with more food. This is achieved by reducing the responsiveness to opportunities for feeding when in the “no food” behavioral pattern, and to switch to highly responsive behavior once food is detected. In this way extensive and intensive search are generated. Thus we observe that the “modularity” of behavior (the two behavioral modes in food and non-food context generated by TODO), provides evolution with structure in which it can generate new specializations (the adaptation of MOVE) and new forms of larger scale information processing (detection patches with more food).

### Relation to foraging theory

Much of foraging theory focuses on foraging efficiency, and uses optimality predictions to assess the foraging behavior of animals (e.g. optimal foraging theory [4], [39], and optimal search theory [9]–[11]). Foraging optima are often specified relative to constraints (e.g. body size, morphology, mode of locomotion, information processing abilities) [4]. However, this does not necessarily give insight into why a species faces particular constraints, since “constraints” are also often evolvable. At present little is known about how constraints arise and change in the evolution of behavior, though presumably this has been a driving factor in the evolution of morphology and information processing abilities (e.g. sensing and cognition). If we assume that in our model the change from restricted to extended decision making represents an evolutionary innovation in information processing, our results show how small evolutionary changes in decision making can lead to a “release from constraints” on a larger scale and shift the system to a new local optima (i.e. going from the bottom to top landscape in Figure 7a). This reveals how the inter-relation between local information processing and larger scale behavioral patterns allows a small increment in memory (i.e. remembering the outcome of a previous search event) to generate a cascade of consequences: (i) differentiation of behavior, (ii) altering the adaptive landscape and eliminating trade-off constraints and (iii) allowing novel foraging specializations.

Such insights are relevant for studying the evolution of cognition, which is likely to involve changes in constraints and behavioral opportunities [40], [41]. Moreover, in light of evolving cognitive complexity our model provides a useful reference. For instance, to establish the impact of elementary spatial cognition such as “remembering where one last found food”, it is probably more appropriate to use TODO-based patch detection as a baseline, rather than random-walks (as in [12]), if individuals can orientate towards food on a local scale without memory. This is also true in terms of model fitting to data to establish mechanisms used by animals during movement. An interesting study by Morales et al. [35] used a spatial grid based model to study movement behavior in elk, assuming that individuals perfectly know the vegetation state of 8 neighboring cells around an individual’s location, and know with less accuracy the state of cells 1 and 2 steps further. Their results show interesting similarities to movement patterns in real elk, and like in our study, shows how orientation to cues in the environment structure movement patterns. However, given the relatively coarse grained resolution of their lattice (28.5 by 28.5 meters), their model does not allow for smaller-scale processes via local visual cues, but assumes spatial cognition. In principle it is possible that if food availability patterns traverse the larger scale grid boundaries of Morales et al.’s model, TODO-based processes could allow individuals to move from grid cell to grid cell according to food availability without using spatial memory. The point here is not to claim the elks couldn’t use spatial memory, but that pattern recognition via TODO could be underestimated. To address this requires models and data with a greater spatial resolution.

Our results also have implications for understanding extensive and intensive search behavior. First, we show that a bi-modal search pattern easily self-organizes from TODO in patchy environments in all evolved specialists whether they evolved there or not. This bi-modal pattern is not an evolved strategy, but simply a reflection of the environment. Bi-modal movement patterns are therefore the default expectation in patchy environments. Secondly, in terms of the extended model, we show how a simple mechanism generating extensive and intensive search modes can be created by evolution. Here there is a difference with the model of Benhamou [12], where bi-modal search is assumed as an adaptive strategy, and studied as a combination of random walks. We find that the regulation of switching between extensive and intensive search does not evolve as a specific strategy in the patchy environment, because it also evolves in the uniform environment (Ext-Uni also shows intensive and extensive search). Instead we find that the specific adaptations in Ext-Patchy function to refine the self-organized extensive and intensive search in order to enhance a new kind of pattern detection: implicitly finding larger patches. This latter pattern detection is not usually considered in foraging theory, but may play an important role in foraging success.

Given the focus of optimal search theory on internally-driven turning strategies [6], [7], [9], [11], it is surprising that we do not find any significant evolution of turning angles. This suggests that in some cases externally-driven turning behavior may pre-empt any need for internally-driven turning strategies and that opportunity-based orientation towards food may be an under-represented aspect in this field [6], [17]. Moreover, we show how individuals can generalize their behavior across environments via TODO, while fixed internally-driven turning strategies are less robust because they need to be specified to a given environment. However, our results depend on the fact that individuals can detect food items from beyond their reach. This may often be the case in animals, but not always, especially if food items are very cryptic. Moreover, given our simplistic implementation of turning behavior, and other model assumptions (e.g. random turning at environment boundary, intermittent searching), more work is needed to specifically address the relationship between internally- and externally-driven turning.

### Conclusions

In terms of the evolution of behavior, the value of our results lie in revealing how small changes in decision making and memory have profound influences on multiple scales relevant for individuals foragers. Clearly our foragers are simplistic (especially cognitively) and therefore it is unlikely that the local optima we find are directly relevant for a given animal species. However, we show that TODO can be a means through which animals could detect larger-scale environmental patterns, which should be taken into account. Moreover we find that extensive search modes can be used to implicitly detect larger food patches in the environment. These findings can be useful to consider when modeling foraging processes and its fitness consequences. Thus our results provide a useful baseline for understanding the evolution of behavioral flexibility and how evolutionary changes in cognition can alter trade-off constraints and adaptive landscapes.

## Supporting Information

Figure S1
**Ancestor traces of evolving foraging parameters in restricted and extended model in patchy and uniform environments.** (a) probability to scan for food again: 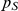 (restricted model), 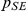, (b) probability to scan for food again after not finding food (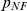) and probability of moving to food (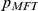) (both only in extended model. (c) probability to move again (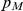), (d) food scan duration (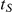), (e) food scan range (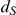), (f) food scan angle (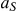), (g) move duration (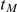), (h) move distance (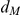), (i) turning angle (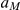). Each dotted line represent lineages from a specific simulation (10 simulations for each model and environment condition).(TIFF)

Figure S2
**Local adaptive landscape in patchy environment of (i) food scan angle (** 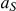 **) and (ii) food scan distance (** 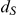 **).** Top: extended model. Bottom: restricted model. From left to right: yearly food intake (fitness), inter-patch travel rate (inverse inter-patch travel time), patch visit time, patch size. Values are normalized within one figure, and a gradient from dark blue to yellow, via green and red, indicates increasing values. Each grid point is the average of 100 samples of a year of foraging. Black circles indicate average evolved parameter values.(TIFF)

Figure S3
**Local adaptive landscape in patchy environment of (i) turning angle (** 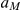 **) and (ii) probability to repeat MOVE (** 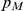 **).** Top: extended model. Bottom: restricted model. From left to right: yearly food intake (fitness), inter-patch travel rate (inverse inter-patch travel time), patch visit time, patch size. Values are normalized within one figure, and a gradient from dark blue to yellow, via green and red, indicates increasing values. Each grid point is the average of 100 samples of a year of foraging. Black circles indicate average evolved parameter values.(TIFF)

Figure S4
**Effect of turning angle (** 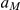 **) on food intake (a) and inter-patch travel distance (b) in Ext-Patchy.** Box plots show median, upper and lower quartiles and whiskers show maximum and minimum values (n = 100 for each box plot). Other parameter values on evolved averages (see Table S1).(TIFF)

Figure S5 **Comparison of evolved specialists in patchy environment.** (a) Patch visit times, (b) inter-patch travel time, (c) average patch size visited. Box plots show median, upper and lower quartiles and whiskers show maximum and minimum values (n = 100 for each specialist). Parameter values set to evolved averages (see Table S1).
(TIFF)

Figure S6 **Comparison of evolved specialists in uniform environment.** (a) Average density of each search event after MOVE, (b) average distance traveled between eat events. Box plots show median, upper and lower quartiles and whiskers show maximum and minimum values (n = 100 for each specialist). Parameter values set to evolved averages (see Table S1).
(TIFF)

Table S1 **Evolved parameter values.** The averages and standard deviations (in brackets) of ancestors between year 800 and 900 of all 10 simulations of all settings (which is approximately 70–80 ancestors per simulation). Those parameters that differ are shown in bold. Angles are shown in degrees, distances in meters and durations in seconds.
(PDF)

Text S1 **Additional file with supplementary information and analysis.** Contents: 1) Model specification choices; 2) Ancestor trace overview; 3) Food scan angle and range; 4) Turning angle and probability to repeat move; 5) Differences between evolved specialists.
(PDF)
