# Supplementary figures and images for: Local Orientation and the Evolution of Foraging: Changes in Decision Making Can Eliminate Evolutionary Trade-offs
Source: PLoS Comput Biol. 2011 Oct 6;7(10):e1002186. doi: 10.1371/journal.pcbi.1002186 (PMC3188503; doi:10.1371/journal.pcbi.1002186)

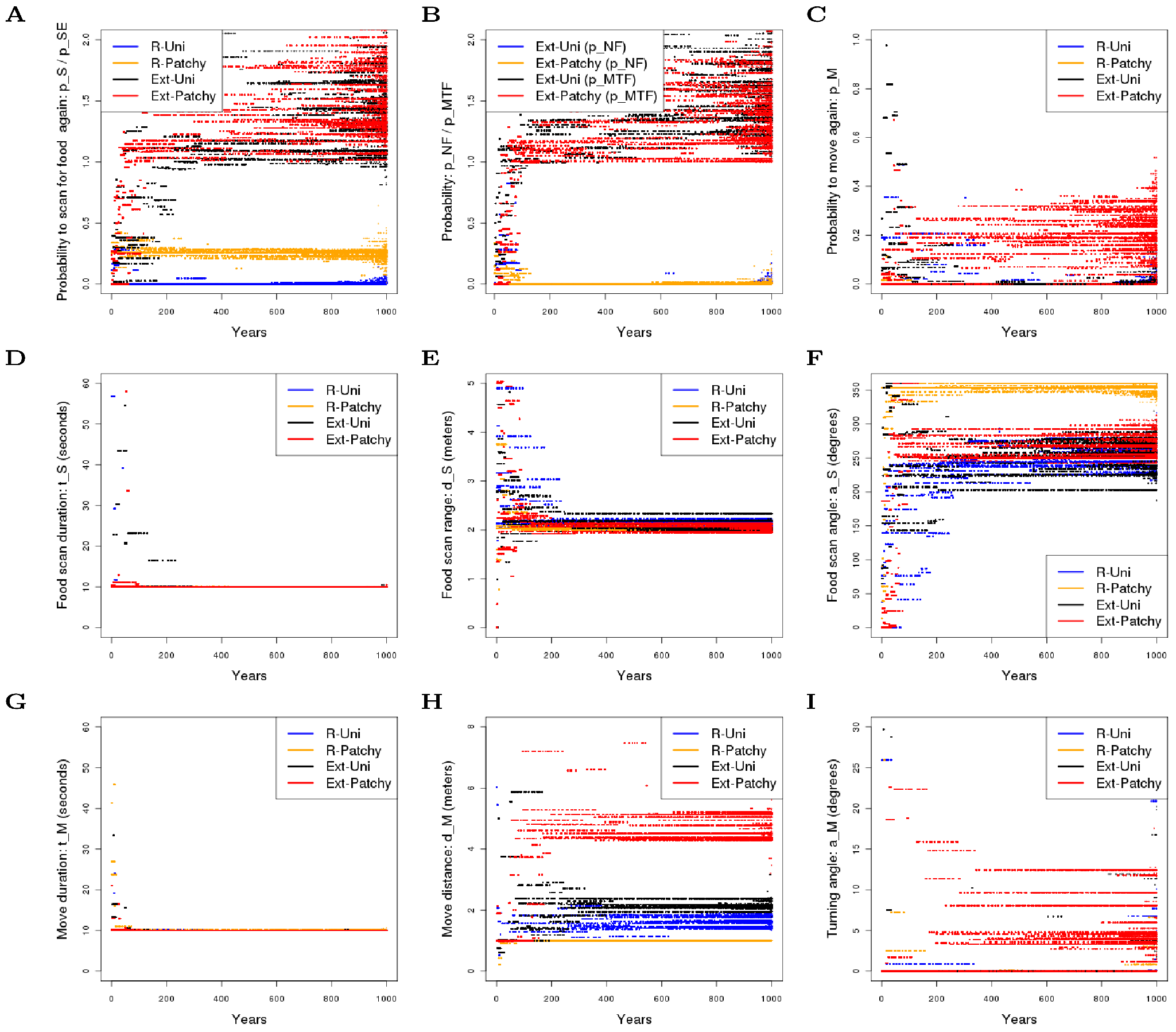

Supplement: Figure S1 — Ancestor traces of evolving foraging parameters in restricted and extended model in patchy and uniform environments. (a) probability to scan for food again: (restricted model), , (b) probability to scan for food again after not finding food () and probability of moving to food () (both only in extended model. (c) probability to move again (), (d) food scan duration (), (e) food scan range (), (f) food scan angle (), (g) move duration (), (h) move distance (), (i) turning angle (). Each dotted line represent lineages from a specific simulation (10 simulations for each model and environment condition). (TIFF) [file pcbi.1002186.s001.tiff]

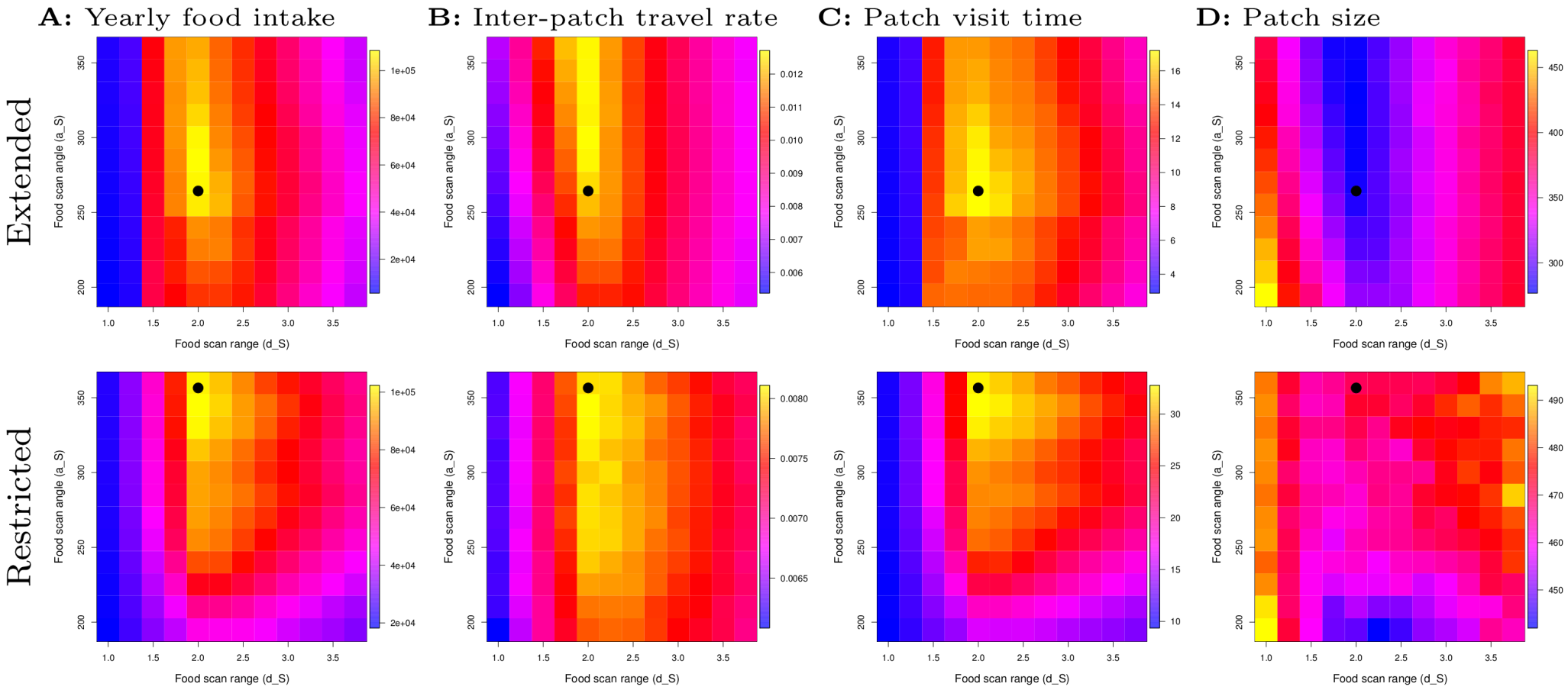

Supplement: Figure S2 — Local adaptive landscape in patchy environment of (i) food scan angle ( ) and (ii) food scan distance ( ). Top: extended model. Bottom: restricted model. From left to right: yearly food intake (fitness), inter-patch travel rate (inverse inter-patch travel time), patch visit time, patch size. Values are normalized within one figure, and a gradient from dark blue to yellow, via green and red, indicates increasing values. Each grid point is the average of 100 samples of a year of foraging. Black circles indicate average evolved parameter values. (TIFF) [file pcbi.1002186.s002.tiff]

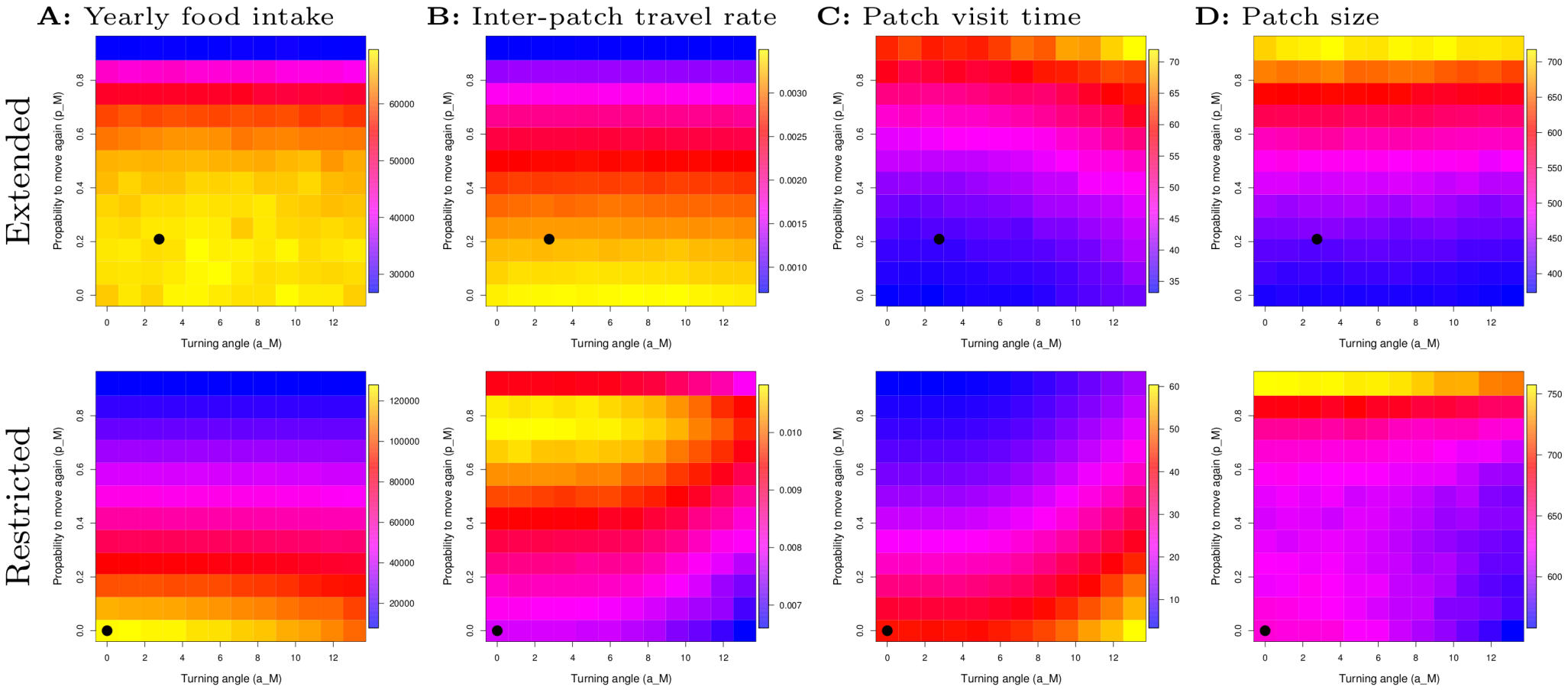

Supplement: Figure S3 — Local adaptive landscape in patchy environment of (i) turning angle ( ) and (ii) probability to repeat MOVE ( ). Top: extended model. Bottom: restricted model. From left to right: yearly food intake (fitness), inter-patch travel rate (inverse inter-patch travel time), patch visit time, patch size. Values are normalized within one figure, and a gradient from dark blue to yellow, via green and red, indicates increasing values. Each grid point is the average of 100 samples of a year of foraging. Black circles indicate average evolved parameter values. (TIFF) [file pcbi.1002186.s003.tiff]

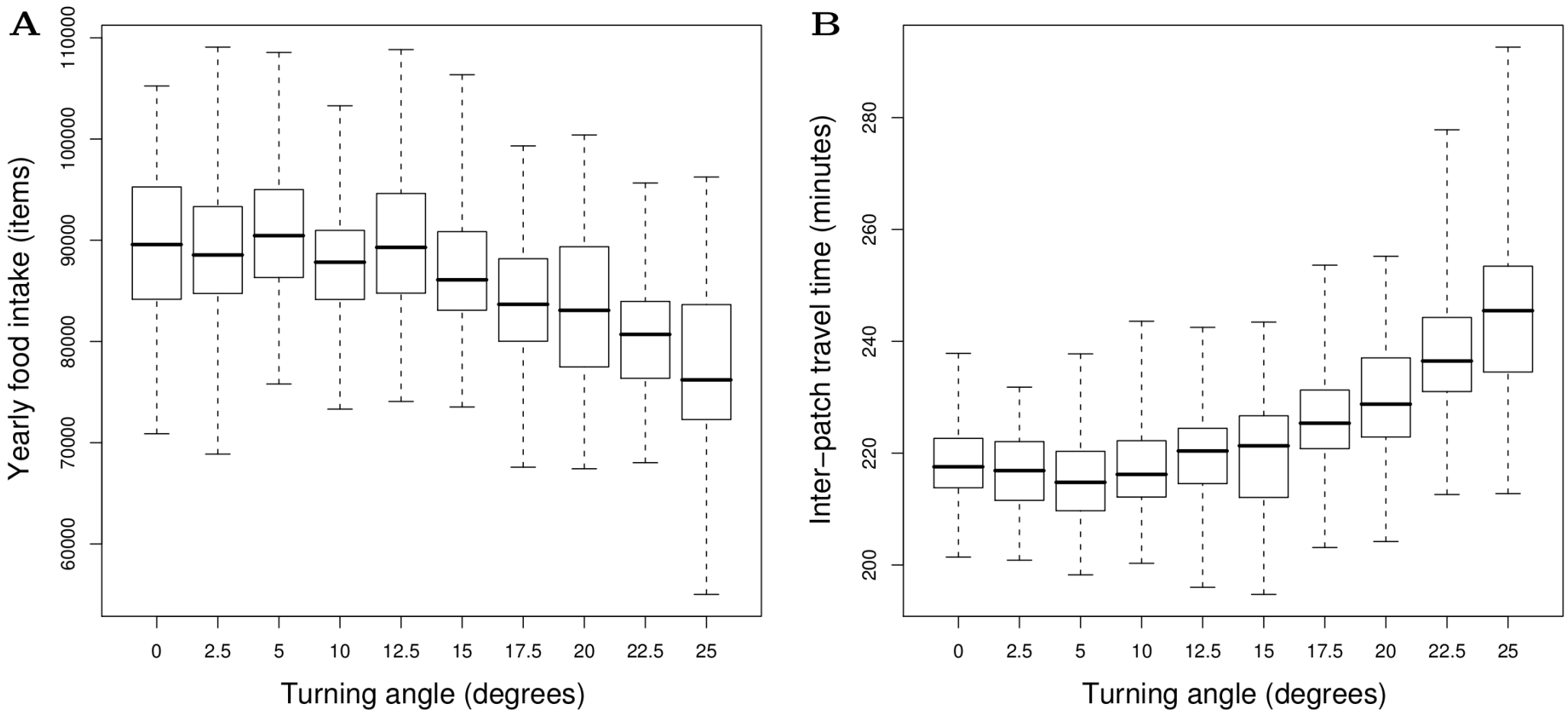

Supplement: Figure S4 — Effect of turning angle ( ) on food intake (a) and inter-patch travel distance (b) in Ext-Patchy. Box plots show median, upper and lower quartiles and whiskers show maximum and minimum values (n = 100 for each box plot). Other parameter values on evolved averages (see Table S1). (TIFF) [file pcbi.1002186.s004.tiff]

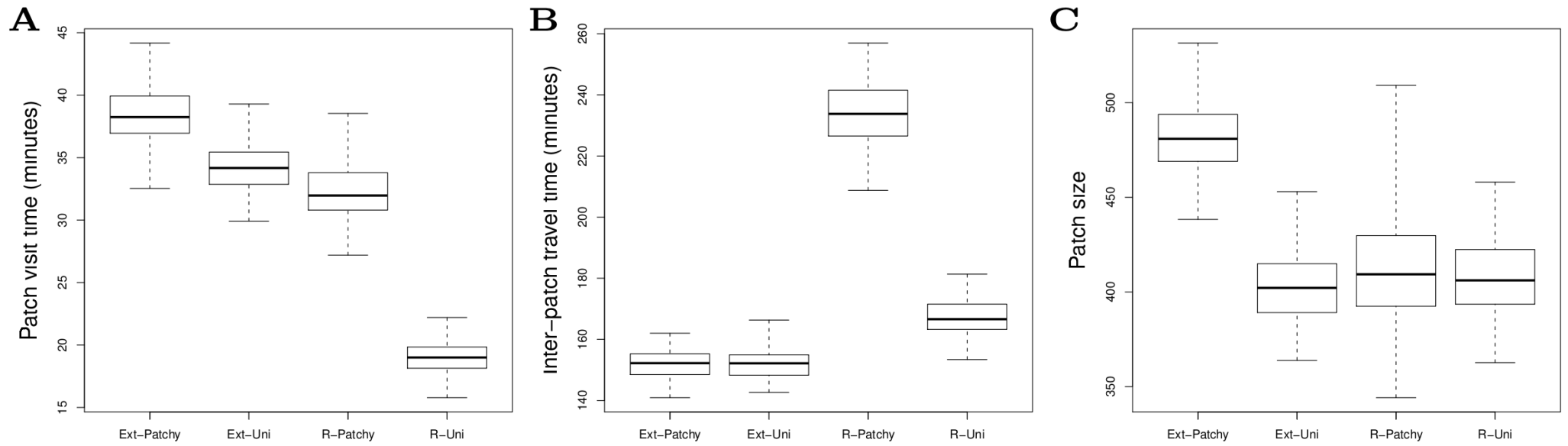

Supplement: Figure S5 — Comparison of evolved specialists in patchy environment. (a) Patch visit times, (b) inter-patch travel time, (c) average patch size visited. Box plots show median, upper and lower quartiles and whiskers show maximum and minimum values (n = 100 for each specialist). Parameter values set to evolved averages (see Table S1). (TIFF) [file pcbi.1002186.s005.tiff]

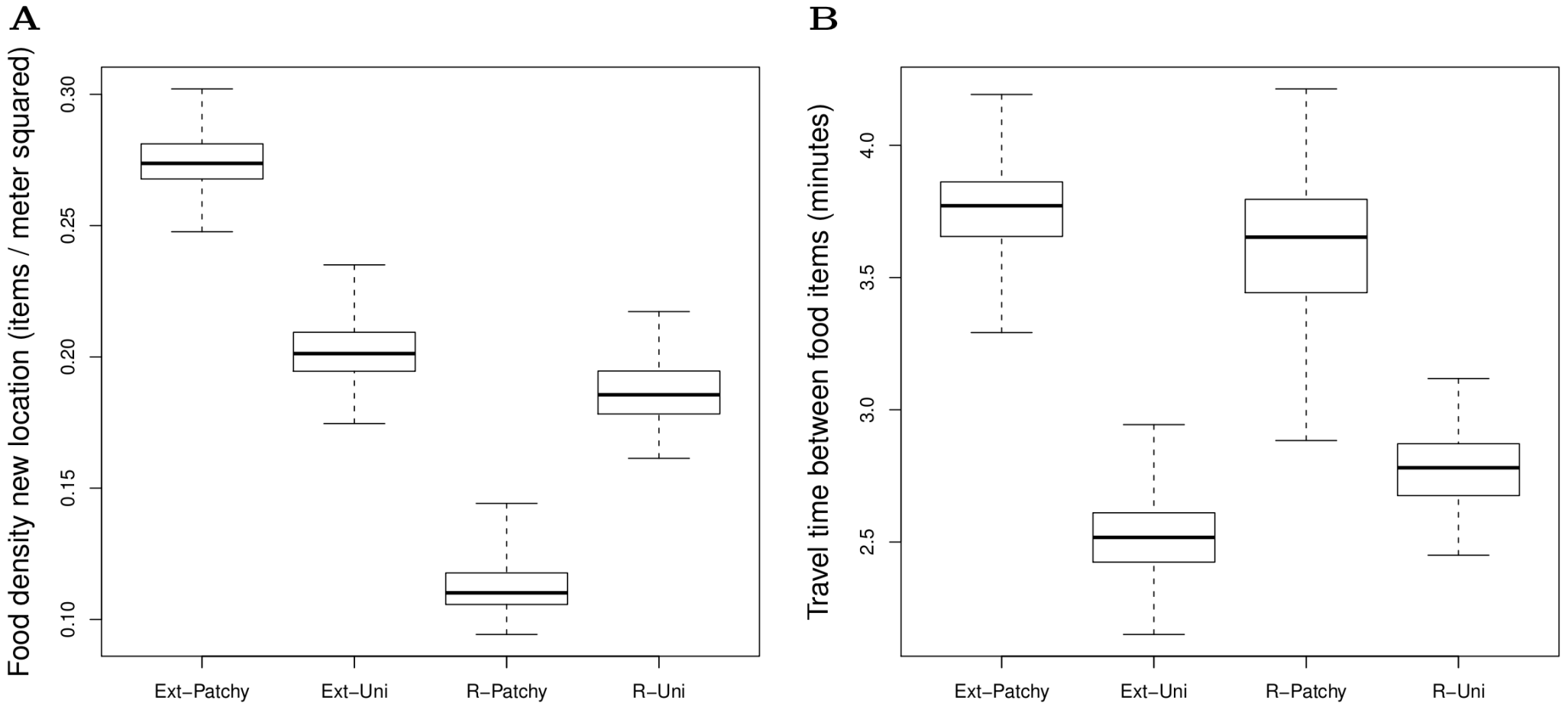

Supplement: Figure S6 — Comparison of evolved specialists in uniform environment. (a) Average density of each search event after MOVE, (b) average distance traveled between eat events. Box plots show median, upper and lower quartiles and whiskers show maximum and minimum values (n = 100 for each specialist). Parameter values set to evolved averages (see Table S1). (TIFF) [file pcbi.1002186.s006.tiff]
