# Supplementary material for: Local Orientation and the Evolution of Foraging: Changes in Decision Making Can Eliminate Evolutionary Trade-offs
Source: PLoS Comput Biol. 2011 Oct 6;7(10):e1002186. doi: 10.1371/journal.pcbi.1002186 (PMC3188503; doi:10.1371/journal.pcbi.1002186)
Supplement: Table S1 — Evolved parameter values. The averages and standard deviations (in brackets) of ancestors between year 800 and 900 of all 10 simulations of all settings (which is approximately 70–80 ancestors per simulation). Those parameters that differ are shown in bold. Angles are shown in degrees, distances in meters and durations in seconds. (PDF) [file pcbi.1002186.s007.pdf]

Table S1: Evolved parameter values

	**Model 1**		**Model 2**	
	Patchy	Uniform	Patchy	Uniform
p_M	0.0 (0.0)	0.0 (0.001)	**0.209 (0.086)**	0.001 (0.007)
t_M	10 (0.01)	10 (0.0)	10 (0.0)	10 (0.0)
d_M	**0.99 (0.002)**	**1.567 (0.156)**	**4.681 (0.353)**	**2.125 (0.099)**
a_M	0.001 (0.182)	0.021 (0.182)	**2.756 (16.108)**	0.295 (9.686)
p_S	**0.251 (0.019)**	0.001 (0.004)	-	-
p_{SE}	-	-	**1.506 (0.333)**	**1.486 (0.429)**
p_{SN}	-	-	0.001 (0.005)	0.0 (0.001)
t_F	10 (0.0)	10 (0.0)	10 (0.0)	10 (0.0)
d_F	2.029 (0.019)	2.115 (0.06)	2.061 (0.069)	2.097 (0.102)
a_F	**356.52 (4.11)**	**248.434 (17.626)**	**264.423 (15.704)**	**253.181 (23.644)**
p_{MTF}	1	1	1.549 (0.428)	1.601 (0.352)
