# Supplementary material for: Local Orientation and the Evolution of Foraging: Changes in Decision Making Can Eliminate Evolutionary Trade-offs
Source: PLoS Comput Biol. 2011 Oct 6;7(10):e1002186. doi: 10.1371/journal.pcbi.1002186 (PMC3188503; doi:10.1371/journal.pcbi.1002186)
Supplement: Text S1 — Additional file with supplementary information and analysis. Contents: 1) Model specification choices; 2) Ancestor trace overview; 3) Food scan angle and range; 4) Turning angle and probability to repeat move; 5) Differences between evolved specialists. (PDF) [file pcbi.1002186.s008.pdf]

Text S1: Supplementary material for "Local Orientation and the Evolution of Foraging: Changes in Decision Making Can Eliminate Evolutionary Trade-offs."

Daniel J van der Post[1,2,3,*], Dirk Semmann[1],
1 Courant Research Center Evolution of Social Behaviour, Georg-August Universität Göttingen, Göttingen, Germany
2 Institute of Artificial Intelligence, University of Groningen, The Netherlands
3 Behavioural Ecology and Self-Organization, University of Groningen, The Netherlands
∗ **E-mail: d.j.vanderpost@gmail.com**

Contents

1. Model specification choices
2. Ancestor trace overview
3. Food scan angle and range
4. Turning angle and probability to repeat move
5. Differences between evolved specialists

1 Model specification choices

See Table 1 in main text for parameters in question.

Spatial and temporal scale: At the minimal level we define the minimal time and spatial scale. A minimal time step of 10 seconds t_{MIN} was chosen to keep the model running fast enough. Space is basically continuous as individuals and resources can be placed at any given location. For convenience we place resource items on intersections of a 1 by 1 meter lattice.

Local information processing: Constraints on movement and perception (local information processing) are defined in terms of the spatial and temporal scale. Maximum speed is basically defined in terms of the minimal time interval, so that the duration of moving 1 meter can never be below t_{MIN}. The maximum range of resource detection (r_D) is then chosen to be small, so that individuals have to move to detect food. Within this range resource detectability is simply maximal, where an item of food can be found during a 10 second FOODSCAN in an area of 10 meters squared (i.e. a search angle of about 270 degrees with a radius of 2 meters). An individuals REACH is then chosen to be below maximal resource detection range. This is quite a reasonable assumption (equal detection range and REACH is probably a limit case) and allows local orientation to food via visual cues.

The following relationship therefore holds: $d_R < r_D$

Energy and life history: Here the minimal time interval sets a constraint on minimal feeding interval t_{MIN_EAT}, namely SEARCH + EAT ($t_{MIN} + t_E$) and for simplicity we set $t_E = t_{MIN}$. t_{MIN_EAT} then sets the maximal energy intake rate in relation to energy per food item E_r and energy metabolism rate E_m: $(E_r/t_{MIN\_EAT}) - E_m$. Moreover, the energy per food item E_r in relation to energy metabolism E_m set the maximum (average) feeding interval individuals can tolerate without net energy loss: $(E_r/t_{MAX\_EAT}) = E_m$ therefore $t_{MAX\_EAT} = E_r/E_m$. The energy required to reproduce $E_M/2$ defines the minimal birth interval: $E_M/2((E_r/t_{MIN\_EAT}) - E_m))$. By setting E_r, E_m and $E_M/2$ in relation to each other ($E_m < E_r <<< E_M/2$), we specify that: individuals must consume in the order of at least 10000 food items to reproduce, within a minimal birth interval of 14 days. Clearly this is quite low for most mammals, but this is never achieved because individuals have to move to find food and global depletion of resources lowers the foraging rate so that birth intervals converge to "life expectancy"

at population carrying capacity. Life expectancy is roughly 10 years due to a death rate of 0.1 per year. This was chosen so that individuals live long enough to experience the full scale of environmental patterns in the environment and can experience multiple reproductive cycles.

Here the following relationships hold: $t_E >= t_{MIN}$, $t_{MIN} + t_E < E_r/E_m$, $E_m < E_r <<< E_M/2$, and $E_M/2((E_r/t_{MIN\_EAT}) - E_m)) >> t_{MAX\_EAT}$ (to ensure reproduction occurs on large enough timescale).

Environment: The environmental settings were chosen to support a viably evolving population and to be able to specify various patchy resource distributions so that animals would have to move through the environment in order to find food. Population size (carrying capacity) is defined in terms of maximum feeding interval in relation to resource influx into the environment: $t_{MAX\_EAT} * R_g$. Thus population size can be directly linked to the ratio E_r/E_m. Resource influx rate gives the total amount of resources per year R_T. The size of the world A then gives the max density of resources R_d. Within the constraints of A and R_T we distribute resources in different ways.

Here the following relationships hold: $t_{MAX\_EAT} * R_g > 100$, $A >> 0$ for space for distributing resources and making movement an issue.

2 Ancestor trace overview

In Figure S1 we show all the ancestor traces of the all the evolving parameters for the different models and different environments. Here we show in more detail the data summarized in Figure 3 in the main text and Table S1.

In terms of decision making we find that (i) in the extended model the probability to scan for food again after finding food always evolves to 1.0 or above (Figure S1a, red and black)(Note that values of more than 1.0 simply give a probability of 1). Concordantly, the probability to scan for food after not finding food evolves to 0.0 (Figure S1b, red and black). In addition, in the extended model, the probability to MOVETOFOOD when food is out of reach evolves to 1.0 or above (Figure S1b, red and black). (ii) In the restricted model the probability to repeat scanning for food only evolves in the patchy environment (Figure S1a, orange, $p_S = 0.25$), while in the uniform environment it does not evolve (Figure S1a, blue). (iii) Repeated moving (p_M) never evolves except in the extended model, patchy environment. However, values do not converge but spread out over a wide range (0.0 - 0.4) (Figure S1c, red). This corresponds to a very flat adaptive landscape indicating a significant degree of neutrality for this parameter (see Figure S3a, top). The same is true for the turning angle (Figure S1i, red).

In terms of behavioral actions we find that (i) in all cases action durations evolve to minimal values (Figure S1d and g) and food scan ranges converge to between 2 and 2.5 meters (Figure S1e). (ii) In all cases food scan angles (a_S) converge in a large range between 200 and 300 degrees, except for the restricted model in the patchy environment where food scan angles are much larger ($345 < a_S <= 360$) (Figure S1f, orange). (iii) Move distance (d_M) evolves to different values. The lowest values are in the restricted model, with strict convergence on $d_M = 1$ in the patchy environment, while in the uniform environment values converge between 1.5 and 2.0 meters (Figure S1h, orange and blue respectively). In the extended model move distances evolve to longer distances both in the uniform environment ($2 < d_M < 2.5$), but more spectacularly in the patchy environment ($4.5 < d_M < 5.5$) (Figure S1h, black and red respectively). (iv) In all cases turning angles converge on 0 (no turning), except in the extended model in the patch environment where they spread over a range between 0.0 and 15.0 degrees (Figure S1i, red lines). Like for p_M we account this to evolutionary drift due to a flat adaptive landscape (see Text S1 section 3).

3 Food scan angle and range

In Figure S2 we show the difference in adaptive landscape for the restricted and extended model in the patchy environment relative to food scan angle (a_S) and food scan range (d_S).

In both cases, yearly food intake peaks at a food scan range between 2-2.5 meters (Figure S2a), which corresponds with evolved values (indicated by black circles). This is the maximum distance at which resources can be detected, and beyond 2 meters is a waste of time and effort: Given a search radius of 2 meters and search time of 10 seconds, the maximum angle to search at which the probability to detect resource items is maximal is:

$$a_F = \frac{2\pi t_F}{\pi(d_F)^2} = \frac{2\pi t_F}{4\pi} = \frac{10}{2} = 5 \quad (1)$$

This means that to maximize resource detection individuals should have $a_F = 5$ radians, or 298.5 degrees. Except for the patch specialist of the restricted model, all specialists have a smaller angle. This is because their search distance is slightly more than 2 meters. Thus a search distance of 2.1 meters requires $a_F = 4.5$ radians or 259.84 degrees, which is around which most specialists converge. Indeed in our analysis, food intake peaks between 250 and 300 degrees (Figure S2a, top). This is also true for the specialists in the uniform environment (results not shown). This is not surprising given the constraints we impose on food detection.

However, food intake peaks at much higher values of food scan angle in the restricted model than in the extended model (Figure S2a, bottom and top respectively). The reason for this is that in the restricted model, individuals need a large scan angle to enhance patch visit times (Figure S2c, bottom). This is not the case in the extended model (Figure S2c, top), because by only using MOVETOFOOD within patches the extended model specialists automatically move from food item to food item enhancing patch visit times. In the restricted model, individuals cannot avoid using MOVE, and hence use a large scan angle to “look back” and return to the patch when inadvertently moving out of it.

Further it is of interest to note that varying food scan ranges and angles changes the sensitivity of individuals to food items (either area searched or probability to detect food items). Here reduced sensitivity can lead to foraging biased to larger patches (Figure S2d). This is similar to the impact of moving longer distances (Figure 7d, top, main text): less sensitivity to specific food items leads to biased foraging in areas with higher density of resources. However, in the case of food scan parameters it also comes at the cost of reduced patch visit times (Figure S2c). Note that this also explains longer inter-patch travel times: less sensitivity means less likelihood to detect a food item, and therefore a patch (Figure S2b).

4 Turning angle and probability to repeat move

In Figure S3 we show the impact of turning angles (a_M) and probability to repeat MOVE (p_M) in the patchy environment. Apparent from yearly food intake (Figure S3a) is that in the extended model (top) there is a much larger range of values for both these parameters for high food intake than in the restricted model (bottom), where food intake is maximal for no turning and no repeated MOVE.

In the restricted model this is the case because repeated MOVE reduces patch visit times (Figure S3c, bottom), and this does not compensate for increased inter-patch travel rate (Figure S3b, bottom), nor finding larger patches (Figure S3d, bottom). Moreover, turning angles are not useful because they reduce inter-patch travel rate (Figure S3b, bottom) and the average size of patch visited (Figure S3d, bottom), and this is not compensated by increased patch visit times (Figure S3c, bottom).

In the extended model, because of the differentiated use of MOVE for inter-patch and MOVETOFOOD within patches, repeated MOVE does not affect patch visit times unless in combination with high turning angles (Figure S3c, top). This means that turning back can still increase patch visit times in the extended model, but this does not weigh up against reduced travel rate (Figure S3b, top). We can also

observe that while repeated MOVE can lead to detection of larger patches by reducing sensitivity to food items encountered (Figure S3d, top), at the same time this leads to more time spent traveling, reducing inter-patch travel rate (Figure S3b, top).

Since turning strategies are intensively studied in optimal search theory, we studied the relative foraging efficiency of a larger range of turning angles more directly by comparing individuals that differ in turning angle in a range from 0 to 25 degrees. This reveals that while initially turning has little impact on food intake rates, that when turns become too large intake rates are reduced (Figure S4a). This corresponds to increasing inter-patch travel time (reduced travel rate) with increased turning (Figure S4b).

This leaves the question: why is there less sensitivity to turning angles in the extended model than in the restricted model? The main reason is the longer move distances in the extended model. This reduces the number of turns per distance covered, and thus effectively reducing the impact of the turning angle parameter. This leads to greater neutrality for this parameter in the extended model patch specialist relative to all other specialists. The general tendency therefore appears to be “move straight”. This avoids previously depleted areas.

5 Differences between evolved specialists

In Figure S5 we show differences of the evolved specialists in the patchy environment to further explain the differences in food intake shown in Figure 6b in the main text. Clearly both patch specialists have greater patch visiting times than R-Uni (Figure S5a). More surprising is that Ext-Uni does at least as well as R-Patchy (Figure S5a). This is due to the fact that the differentiation of movement behavior within and between patches allow extended model specialists to enhance patch visit times.

This also means that both extended model specialists move rapidly between patches (Figure S5b). In contrast R-Patchy has the slowest inter-patch travel rate (Figure S5b), which is a direct indication of the trade-off faced in the restricted model: to stay longer in patches, inter-patch travel is sacrificed.

Finally we note that the difference between Ext-Patchy and Ext-Uni in terms of food intake (see Figure 6b in main text) does not clearly come to expression from differences in inter-patch travel, nor patch visiting times (although the Ext-Patchy has somewhat longer visits than Ext-Uni, Wilcoxon rank sum test, $p < 0.0001$). However the size of patches visited by Ext-Patchy are larger (Figure S5c, Wilcoxon rank sum test, $p < 0.0001$). This effect is caused by the longer move distance of Ext-Patchy allowing it to bias its patch visiting to larger patches. By skipping smaller patches on the way it manages to find larger patches in the same time it takes Ext-Uni to find somewhat smaller patches.

In Figure S6 we show further differences of the evolved specialists in the uniform environment, next to food intake shown in Figure 6a in the main text. The different movement behavior of the different specialists has a large impact of the density of resources they experience each food scan event (Figure S6a). Ext-patchy moves long distances and ends up searching for food where there are greater densities of food. In contrast R-Patchy moves such short distances and repeats food scans that it often scans areas it has just depleted.

Both uniform specialists are intermediate to these extremes as a consequence they spend the least time traveling between the food items they find (Figure S6b). Here Ext-Patchy spends more time because it skips food items on the way by moving too far, while R-Patchy spends more time because it has more redundant search.
